# A Wilson–Cowan reservoir computer for interpretable spatiotemporal vision

**DOI:** 10.1038/s41598-026-49359-5

**Published:** 2026-05-04

**Authors:** Sharmarke A. Gabayre, Sergey Savel’ev, Varuna De Silva, Mindula Illeperuma, Xiyu Shi

**Affiliations:** 1 Institute of Digital Technologies, Loughborough University London, 3 Lesney Avenue, Here East, Queen Elizabeth Olympic Park, E20 3BS London, UK; 2 Department of Physics, Loughborough University, Epinal Way, LE11 3TU Loughborough, UK

**Keywords:** Wilson–Cowan Model, Reservoir Computing, Neural Wave Dynamics, Spatio-Temporal Processing, Biologically Plausible Neural Networks, Visual Cortex Modelling, Structured Neural Reservoirs, Engineering, Mathematics and computing, Neuroscience

## Abstract

We present a Wilson–Cowan reservoir computer (WC–RC) that treats a retinotopic excitatory–inhibitory neural field as a structured reservoir. Travelling waves and bounded oscillations provide an interpretable spatiotemporal basis, while a two-stage sampler (40 sites $\times$ 200 steps) exports 8000 features per input—a $>99\%$ reduction with unchanged integration cost. On MNIST and Fashion-MNIST, recurrent readouts trained on these features achieve strong performance within this fixed export budget: Att-LSTM reaches $85.4\%/79.0\%$, while GRU and vanilla LSTM yield similar accuracies (all with tight Wilson 95% confidence intervals). A simple MLP readout performs markedly worse ($45.6\%/54.2\%$), whereas a compact ridge classifier still attains non-trivial performance ($71.2\%/72.7\%$), indicating that the exported WC–RC representation is partially linearly decodable but benefits further from temporal modelling. Selective suppression of lateral couplings ($\lambda \in \{0,0.25,0.5,0.75,1\}$) shows that reinstating wave dynamics improves recurrent models while degrading the MLP, supporting a functional role for propagating dynamics. A complementary neighbour-coupling ablation, implemented via a diffusion-like scaling factor $\psi$, shows that increasing lateral spread beyond the baseline regime reduces late-time spatial variance and degrades recurrent-readout accuracy, indicating that useful computation depends on balanced wave dynamics rather than maximal smoothing. At matched exported-feature and readout budgets, ESN baselines underperform ($\approx 64\%/\approx 73\%$), whereas compact CNNs achieve higher accuracy but with larger parameter and MAC budgets and without wave interpretability. Supplementary analyses confirm numerical fidelity and relate performance gains to propagation coherence. We outline a fixed-point streaming-convolution mapping for FPGA/ASIC deployment, positioning WC–RC as an interpretable, energy-aware reservoir for neuromorphic vision.

## Introduction

Neuromorphic computing departs from conventional Artificial Intelligence (AI) by prioritising *energy efficiency*, *locality* and *biological plausibility*^1^. Among population models with strong biological grounding, the Wilson–Cowan (WC) framework is foundational for capturing excitatory–inhibitory (E–I) interactions and their spatio-temporal consequences^2–4^. WC neural fields reproduce oscillations and travelling waves observed in cortex^5–7^, offering a mechanistic route to temporally structured computation often absent from simplified neuromorphic abstractions^8,9^.

*Reservoir computing* (RC) formalises the idea that a fixed or lightly plastic recurrent substrate transforms inputs into a high dimensional dynamical basis, with learning confined to a lightweight readout^10–14^. RC has been realised across quantum, spintronic and wave-based substrates^15–18^, yet two issues arise from a biological and systems standpoint: device-level or spiking reservoirs may lack population-level plausibility^19–21^, and random connectivity reduces interpretability and hampers principled hardware co-design^13^. We therefore compare a structured WC reservoir against a random echo-state network (ESN) baseline and compact CNNs under matched I/O and reporting budgets (MNIST and Fashion-MNIST), to isolate the contribution of structured waves versus random dynamics or purely feed-forward computation.

**Fig 1:**
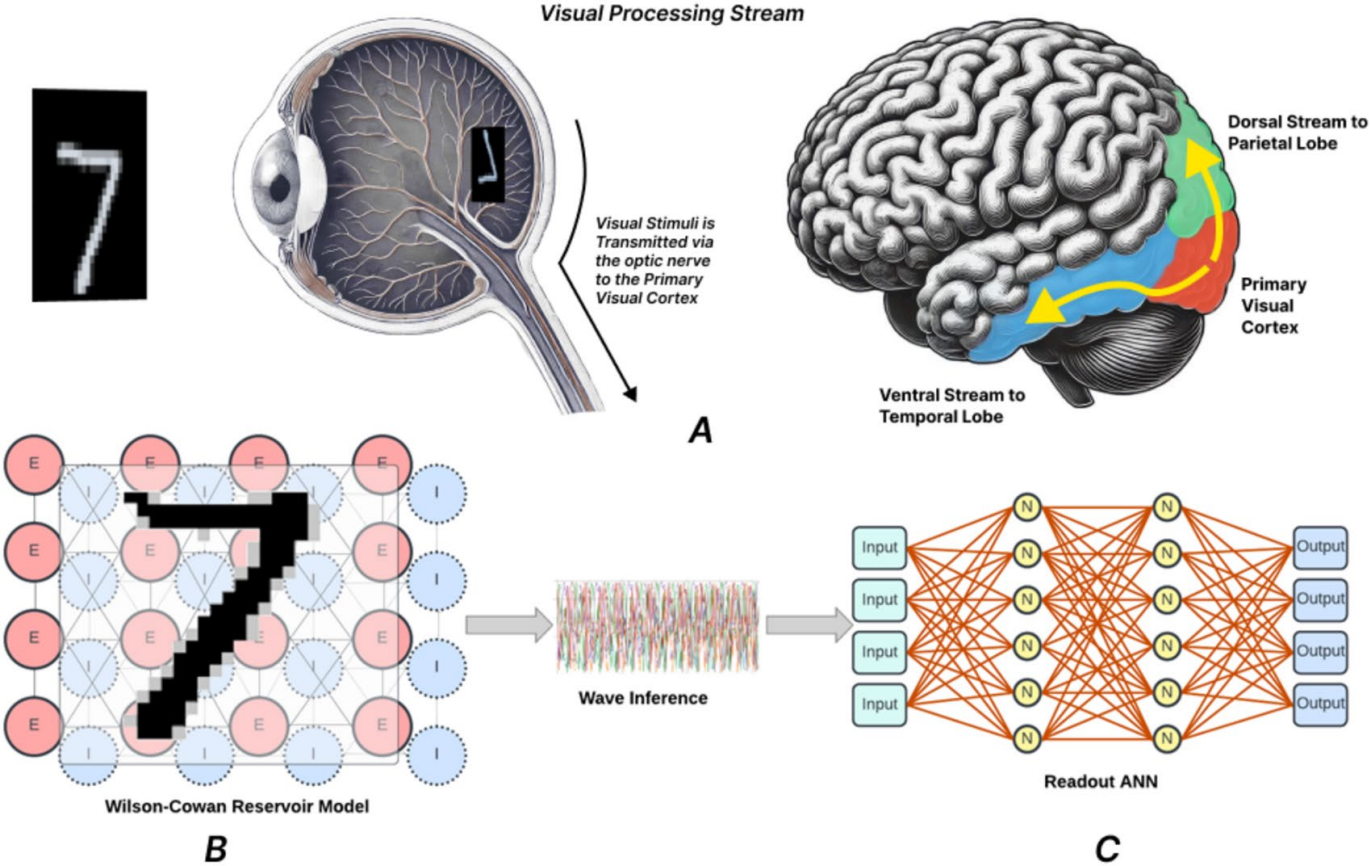
(**A**) Mid-level visual pathway: retinal input drives retinotopic fields exhibiting waves/oscillations. (**B**) WC–RC substrate: a 2D E–I lattice with local lateral couplings produces travelling-wave transients under image drive. (**C**) Readout: compact spatio-temporal samples feed a lightweight classifier; energy, latency and calibration are reported alongside accuracy.

We hypothesise that early visual cortex functions as a *structured reservoir*: a retinotopic E–I neural field whose wave dynamics provide a rich, partially linearly decodable basis for mid-level vision that is further improved by temporal readouts. We instantiate this view with a Wilson–Cowan reservoir computing (WC–RC) architecture that embeds biologically motivated lateral couplings on a two-dimensional (2D) lattice (Fig. 1b). In contrast to random echo state networks^22^ or purely event-driven spiking stacks^9^, our substrate exploits *continuous* nonlinear wave propagation across a structured E–I grid, producing oscillatory transients and wave interference patterns consistent with cortical phenomenology^5,7,23^. Because the WC update decomposes into a small set of local spatial kernels plus leaky integration, it maps naturally to streaming convolution hardware; this affords an *energy-aware* route to deployment that aligns with neuromorphic objectives^1,24^. For completeness on efficient readouts, we also position our approach alongside low-bit/binary neural network literature^25–28^, which provides practical pathways for quantised WC–RC heads. Recent neuromorphic prototypes demonstrate that structured, locality-preserving updates can be realised efficiently in hardware, reinforcing the case for field-style substrates that map to streaming computation^1,24,29^.

We treat energy and compute as first-class outcomes alongside accuracy, confidence intervals, ablations and model complexity comparisons. Specifically, we report multiply–accumulate (MAC) counts for readouts, exported-feature budgets, and measured wall-time for reservoir integration; for the reservoir we provide transparent accounting per timestep (stencil taps and pointwise non-linearities) rather than relying on closed-form MAC formulae that are hardware-dependent. We then analyse accuracy–budget trade-offs, linear versus recurrent decoding, and the effects of wave suppression, graded modulation ($\lambda$-sweeps), and diffusion-like neighbour-coupling changes ($\psi$-ablation), following recent RC work emphasising efficient physical substrates^12,14^. This clarifies where wave-mediated context is beneficial per unit compute, not just in absolute accuracy.

Our contributions are as follows:

1. Cortex-as-reservoir formalisation. We cast a WC neural field as a *structured reservoir* for vision, identify bounded oscillatory regimes (fading memory), and show that field states are partially linearly decodable at a fixed readout budget, with further gains obtained from recurrent temporal decoding and a compact ridge-classifier baseline.
2. Waves versus no waves, quantitatively. We introduce a fully suppressed control (neighbour bands set to zero) and *graded* E–I modulation via $\lambda$-scaling of lateral couplings. Across MNIST and Fashion-MNIST, recurrent heads benefit from non-zero $\lambda$ while MLP probes do not, establishing the functional role of travelling waves/oscillations.
3. Balanced propagation rather than maximal smoothing. Through a complementary diffusion-like neighbour-coupling ablation ($\psi$-scaling), we show that increasing lateral spread beyond the baseline regime reduces late-time spatial variance and degrades recurrent-readout accuracy, indicating that useful computation depends on balanced wave dynamics rather than maximal blur.
4. Structured WC–RC vs random reservoirs and compact CNNs. At matched exported-feature budgets and training protocol, we compare WC–RC heads against an ESN baseline and two tiny CNNs. CNNs remain strongest in raw accuracy; WC–RC offers controllable, interpretable dynamics with competitive performance against ESN and clear gains over no-wave controls.
5. Readout economy with explicit accounting. Spatial tiling and temporal sub-sampling yield fixed exported-feature budgets; we report readout MACs and reservoir run-time, and we examine accuracy as a function of exported budget (sites$\times$timesteps).
6. Stability-aware numerics and quantisation pathway. We compare Euler/Heun/RK4 under fixed error tolerances, report stable operating steps/horizon, and discuss fixed-point deployment of readouts, contextualised by low-bit/binary methods^25–28^.

## Related works

Neuromorphic computing has progressed rapidly under pressures for adaptability, energy efficiency, and biological plausibility^1^. Within this landscape, RC provides a principled separation between a (largely) fixed recurrent substrate and a lightweight trainable readout^10–12,14^. Classical implementations, notably ESNs, achieve high-dimensional temporal projection with random recurrent weights and readout-only learning^13,22^. However, random connectivity reduces interpretability and weakens alignment with cortical organisation, while device- or spike-level implementations can underplay population-level phenomena that are prominent in cortex^8,9,19^. In this work we therefore benchmark a structured WC reservoir against an ESN and compact CNNs under matched I/O and reporting budgets (MNIST and Fashion-MNIST), to separate the value of wave-mediated structure from random reservoirs and purely feed-forward pipelines.

Beyond abstract RNNs, RC has been instantiated in a variety of physical substrates, including quantum^15^, spintronic^16^, wave-scattering media^17^, and emerging in-sensor or memristive platforms^19,21^. While these systems demonstrate impressive efficiency, they often trade biological fidelity for engineering convenience. For instance, feature expansion via multiplicative (Sigma–Pi) interactions can improve prediction on multivariate time series but complicates scaling and hardware realisation^30^. In contrast, structured reservoirs with spatially organised connectivity^13^ and hardware-friendly streaming computation^12,24^ offer a path to reproducible, energy-aware designs. Complementarily, low-bit/binary neural networks provide practical routes to efficient readouts and probes^25–28^, which we discuss as deployment options for WC–RC heads.

**Wilson–Cowan fields as biologically grounded reservoirs.** The Wilson–Cowan (WC) framework furnishes a rigorous population model of excitatory–inhibitory (E–I) interactions^2,3^, with extensions to hierarchical and spatially structured settings^4^. WC neural fields reproduce oscillations and travelling waves observed in cortex^5–7^ and have been used to model distributed computation and wave interference in visual circuits^23^. This stands in contrast to many SNN pipelines that emphasise spike timing but abstract away continuous wave propagation at the population level^9^. Related oscillator-based RC studies (e.g., self-sustained local networks) show that structured, spatially constrained dynamics can support useful temporal computation^31^, but typically retain unstructured plasticity or connectivity rules that hinder hardware co-design. Our formulation instead fixes lateral kernels and then *systematically modulates* them through fully suppressed controls, graded $\lambda$-sweeps, and complementary diffusion-like neighbour-coupling ablations to quantify the functional role of waves.

### Theoretical and practical implications

#### From random transients to wave-mediated context

Prevailing RC paradigms often rely on random connectivity and, in some cases, operation near the “edge of chaos” to maximise separability and memory^13,14^. By contrast, a WC-based reservoir leverages *constructive interference of neural waves* and bounded oscillations to furnish a rich yet interpretable temporal basis aligned with cortical beta/gamma phenomena^5,7^. This shift replaces opaque randomness with biologically motivated structure, enabling analysis via stability and dispersion tools (e.g., bounded echo regimes) and facilitating linear decodability tests central to RC^10^. Our experiments explicitly relate controlled changes in wave strength to downstream performance in order to establish function rather than epiphenomenon.

#### Interpretability and hardware co-design

Structured spatial sampling and retinotopic lateral kernels narrow the “interpretability gap” by tying representational changes to measurable wave metrics (propagation coherence, dominant-band concentration)^8^. Practically, the WC update decomposes into a small set of local spatial kernels plus leaky integration, which maps to fixed-point, streaming-convolution hardware and aligns with recent neuromorphic demonstrations of efficient spatiotemporal compute^1,12,24,29^. Rather than a hardware-specific MAC formula, we report transparent per-timestep stencil/activation counts and exported-feature budgets, and we consider quantised readouts in light of the binarisation literature^25–28^. This makes energy a first-class outcome (joules/decision) alongside accuracy and calibration, an increasingly emphasised perspective^1,14^.

#### Limitations and prospects

**Fig 2:**
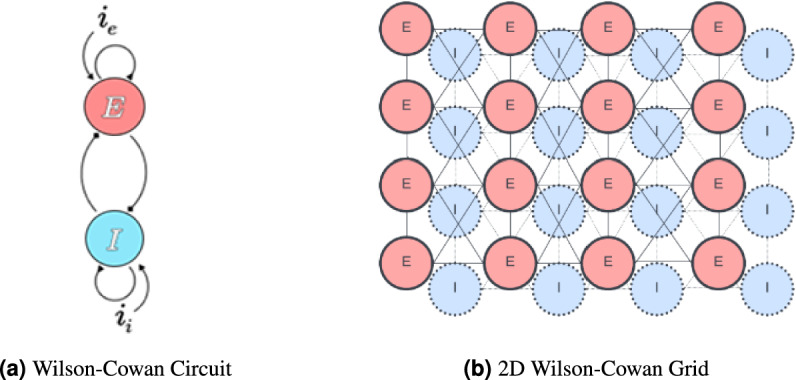
(**A**) The basic Wilson-Cowan circuit consists of two interconnected cells: an excitatory cell (E) and an inhibitory cell (I). Both cells have feedback loops that influence their own activity. Arrows and diamond-junctions on the connecting lines indicate excitatory and inhibitory connections, respectively. (**B**) The generic circuit is then used as a matrix that is distributed to match the dimensionality of the input images (28x28).

A fixed-weight WC reservoir omits synaptic plasticity that is central to biological learning. Local, inhibitory or homeostatic plasticity rules offer a biologically plausible route to adaptive substrates without sacrificing stability^7,32^. In parallel, photonic and memristive implementations may benefit from the WC paradigm’s locality and determinism^19,33^, providing a reproducible path to low-latency, low-power mid-level vision that remains interpretable and testable under reservoir criteria (Fig. 2).

## Methodology

### Wilson–Cowan Reservoir Computing (WC–RC)

**Fig 3:**
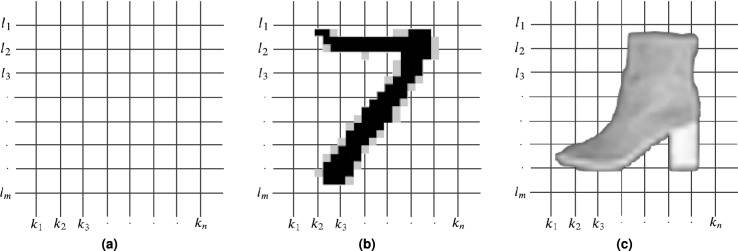
(**A**) Schematic of a 2D lattice; rows $l_1,\dots ,l_m$, columns $k_1,\dots ,k_n$. (**B**, **C**) The same lattice overlaid with an MNIST digit and FMNIST image; darker pixels indicate stronger stimulus drive.

The Wilson–Cowan (WC) framework models reciprocal dynamics between excitatory and inhibitory neural populations and has long served as a canonical description of cortical population activity^2,3^. Its ability to reproduce oscillations and travelling waves observed in cortex makes it a natural substrate for temporally structured computation^5,23^. We instantiate a *structured reservoir* by arranging WC populations on a two-dimensional (2D) retinotopic lattice whose spatial dimensions match the input ($28{\times }28$ for MNIST/FMNIST), forming the Wilson–Cowan Reservoir Computer (WC–RC). Each lattice site hosts an excitatory population $r_E$ and an inhibitory population $r_I$, allowing spatially local interactions to give rise to wave-like dynamics (Fig. 3A, B, C). We validate this model’s parameters in the Supplementary Section A.

A static or time-varying visual input is represented by a function $image(t,l,k)$, and the population activities evolve as

1 $$\begin{aligned} \begin{aligned} \tau _E \frac{\partial r_E(t,l,k)}{\partial t} = -r_E(t,l,k) + \sigma (A_E(t,l,k)), \\ \tau _I \frac{\partial r_I(t,l,k)}{\partial t} = -r_I(t,l,k) + \sigma (A_I(t,l,k)), \end{aligned} \end{aligned}$$

with time constants $\tau _E,\tau _I$ and a saturating nonlinearity $\sigma (\cdot )$. The total inputs $A_E, A_I$ collect self-feedback and external drive:

2 $$\begin{aligned} \begin{aligned} A_E&= w_{EE}^0 r_E(t,l,k) - w_{EI}^0 r_I(t,l,k)+ w_{EE}^1 \sum r_E^{(hv)}(t,l,k) - w_{EI}^1 \sum r_I^{(hv)}(t,l,k) \\&\quad + w_{EE}^2 \sum r_E^{(d)}(t,l,k) - w_{EI}^2 \sum r_I^{(d)}(t,l,k)+ j_E(t,l,k), \end{aligned} \end{aligned}$$

3 $$\begin{aligned} \begin{aligned} A_I&= w_{IE}^0 r_E(t,l,k) - w_{II}^0 r_I(t,l,k)+ w_{IE}^1 \sum r_E^{(hv)}(t,l,k) - w_{II}^1 \sum r_I^{(hv)}(t,l,k) \\&\quad + w_{IE}^2 \sum r_E^{(d)}(t,l,k) - w_{II}^2 \sum r_I^{(d)}(t,l,k)+ j_I(t,l,k), \end{aligned} \end{aligned}$$

where superscripts $(hv)$ and $(d)$ denote immediate horizontal/vertical and diagonal neighbours, respectively. Zero Dirichlet boundaries are applied:

4 $$\begin{aligned} r_{E}(t,l\le 0,k) = r_{E}(t,l>m,k) = r_{E}(t,l,k\le 0) = r_{E}(t,l,k>n) = 0, \end{aligned}$$

5 $$\begin{aligned} r_{I}(t,l\le 0,k) = r_{I}(t,l>m,k) = r_{I}(t,l,k\le 0) = r_{I}(t,l,k>n) = 0, \end{aligned}$$

6 $$\begin{aligned} \begin{aligned} \text {image}(t, l \le 0,~k)&= \text {image}(t, l> m,~k) = \text {image}(t,~l,~k \le 0) = \text {image}(t,~l,~k > n) = 0, \end{aligned} \end{aligned}$$

with initial state $r_E(t{=}0,~l,~k)= r_I(t{=}0,~l,~k)=0$. The input coupling is

7 $$\begin{aligned} \begin{aligned} j_E = \alpha \cdot \text {image}(t,~l,~k),\qquad j_I = (1-\alpha )\cdot \text {image}(t,~l,~k), \end{aligned} \end{aligned}$$

and we collect weights into weight matrices:

8 $$\begin{aligned} \begin{aligned} W^0 = \begin{bmatrix} w_{EE}^0 & w_{EI}^0 \\ w_{IE}^0 & w_{II}^0 \end{bmatrix}, \quad W^1 = \begin{bmatrix} w_{EE}^1 & w_{EI}^1 \\ w_{IE}^1 & w_{II}^1 \end{bmatrix}, \quad W^2 = \begin{bmatrix} w_{EE}^2 & w_{EI}^2 \\ w_{IE}^2 & w_{II}^2 \end{bmatrix}. \end{aligned} \end{aligned}$$

This construction yields a retinotopic neural field with local E–I couplings whose transients (oscillations, travelling waves) provide a high-dimensional temporal basis amenable to readout, aligning with a “structured reservoir” view of cortex^5,10^.

### Simulation procedure

#### Inputs and normalisation

External drives $j_E,~j_I$ are derived from pixel intensities linearly normalised to $[0,1]$. In addition to static images (MNIST), we consider simple dynamic stimuli (e.g., moving edges) to probe temporal responses, with optional Gaussian pre-smoothing to suppress pixel-level artefacts.

#### Initialisation and geometry

States $r_E,~r_I$ are initialised to zero across the grid. The lattice size matches the input (e.g., $28{\times }28$ for MNIST; $12{\times }12$ for reduced experiments).

#### Integration

We simulate $t=2000$ steps with fixed $\Delta t=0.01$ using classical fourth-order Runge–Kutta (RK4), implementing the standard four-slope update as follows:

- Step 1: Compute initial slope ($k_1$)
  9 $$\begin{aligned} \begin{aligned} k_1^E&= f_E(r_E,r_I,t) = \frac{-r_E+\sigma (A_E)}{\tau _E}, \\ k_1^I&= f_I(r_E,r_I,t) = \frac{-r_I+\sigma (A_I)}{\tau _I}. \end{aligned} \end{aligned}$$
- Step 2: Compute intermediate slopes ($k_2$, $k_3$) at half timestep
  10 $$\begin{aligned} \begin{aligned} k_2^E&= f_E\left( r_E + \frac{\Delta t}{2}k_1^E, r_I + \frac{\Delta t}{2}k_1^I, t + \frac{\Delta t}{2}\right) , \\ k_2^I&= f_I\left( r_E + \frac{\Delta t}{2}k_1^E, r_I + \frac{\Delta t}{2}k_1^I, t + \frac{\Delta t}{2}\right) , \\ k_3^E&= f_E\left( r_E + \frac{\Delta t}{2}k_2^E, r_I + \frac{\Delta t}{2}k_2^I, t + \frac{\Delta t}{2}\right) , \\ k_3^I&= f_I\left( r_E + \frac{\Delta t}{2}k_2^E, r_I + \frac{\Delta t}{2}k_2^I, t + \frac{\Delta t}{2}\right) . \end{aligned} \end{aligned}$$
- Step 3: Compute final slope ($k_4$) at full timestep
  11 $$\begin{aligned} \begin{aligned} k_4^E&= f_E(r_E + \Delta t \cdot k_3^E, r_I + \Delta t \cdot k_3^I, t + \Delta t), \\ k_4^I&= f_I(r_E + \Delta t \cdot k_3^E, r_I + \Delta t \cdot k_3^I, t + \Delta t). \end{aligned} \end{aligned}$$
- Step 4: Update neuron states using the slopes
  12 $$\begin{aligned} \begin{aligned} r_E(t + \Delta t)&= r_E(t) + \frac{\Delta t}{6}(k_1^E + 2k_2^E + 2k_3^E + k_4^E), \\ r_I(t + \Delta t)&= r_I(t) + \frac{\Delta t}{6}(k_1^I + 2k_2^I + 2k_3^I + k_4^I). \end{aligned} \end{aligned}$$

#### Recorded signals

We store full spatiotemporal states, yielding a tensor of shape $H\times W\times 2\times T$ (e.g., $28\times 28 \times 2 \times 2000$ for MNIST). For global summaries we compute the spatial sum of $r_E$ over time (a 1D signature), and for spatial selectivity we compare terminal $r_E$ maps to input structure (MSE, Pearson correlation).

#### Operating regimes and parameterisation

Unless stated otherwise, inhibitory and excitatory time constants are set to $\tau _I{=}2.0$ and $\tau _E\in [1.0,\,3.0]$; the input split $\alpha \in [0.4,\,0.7]$. We report settings that fall in bounded, oscillatory regimes validated numerically (Supplementary A), and we ablate $\tau _E$ and $\alpha$ where relevant to show robustness of downstream readouts.

#### Wave–suppression protocol ($\lambda$-sweep)

To quantify the functional role of lateral propagation, we scale neighbour couplings by a scalar $\lambda \in [0,1]$:

13 $$\begin{aligned} \big (w^{1}_{XY},\,w^{2}_{XY}\big )\;\leftarrow \;\lambda \,\big (w^{1}_{XY},\,w^{2}_{XY}\big )\quad \text {for }X,Y\in \{E,I\}, \end{aligned}$$

leaving on-site terms $W^{0}$ unchanged. Thus $\lambda {=}0$ removes horizontal/vertical and diagonal interactions (no waves), and $\lambda {=}1$ restores the full field. For each $\lambda$ we generate feature sets from identical image splits, enabling like-for-like comparisons of accuracy and confidence intervals.

#### Diffusion-like neighbour-coupling ablation ($\psi$-scaling)

To test whether stronger lateral spread alters late-time field structure and downstream utility, we introduce a complementary ablation in which neighbour couplings are scaled by $\psi \in \{1.00,\,1.25,\,1.75\}$:

14 $$\begin{aligned} W^{1}\leftarrow \psi W^{1},\qquad W^{2}\leftarrow \psi W^{2}, \end{aligned}$$

while $W^{0}$ is held fixed. Here $\psi {=}1.00$ denotes the baseline reservoir, and larger $\psi$ values strengthen diffusion-like spatial spread. For each setting we compute late-time spatial variance of the excitatory field and retrain the same readout families on exported features, allowing us to assess whether stronger smoothing preserves or degrades task-relevant structure.

#### Readout preparation (for classification)

For classification experiments, the reservoir is used as a feature generator. We extract compact features via structured spatiotemporal sampling: $40$ lattice sites (20 $E$+20 $I$) sampled across $200$ timesteps (every 10 steps), yielding an $8000$-dimensional sequence per image. These sequences are provided to lightweight readouts (LSTM, GRU, attention-augmented LSTM, MLP, and ridge regression) trained with fixed hyper-parameters; details and splits are shared across MNIST and Fashion-MNIST to enable matched comparisons.

#### Baselines and budget matching

We include (i) an ESN with spectral-radius-controlled random reservoir and matched exported-feature/readout budgets, and (ii) compact CNN baselines (TinyCNN-A/B) sized for similar parameter/MAC envelopes. We include this strictly to maintain experimental rigour, facilitating valid comparisons with random reservoirs and small CNNs under identical budgetary constraints.

#### Energy and implementability hooks

Although simulations are performed in floating point, the WC update decomposes into six local spatial kernels plus leaky integration per step, which maps to streaming-convolution hardware in fixed point. We therefore report multiply–accumulate counts (MACs/step $=6k^2N^2$), exported-feature sizes, and simple latency proxies. This component-wise accounting mirrors recent neuromorphic implementations that separate update stencils, state I/O and readout costs^1,12,24,29^.

#### Task demonstration

On MNIST and Fashion-MNIST, normalised digit images drive the reservoir; the resulting dynamics provide features for downstream classifiers. This establishes the feasibility of combining biologically interpretable WC dynamics with the reservoir paradigm for mid-level vision, and supports controlled ablations linking wave strength and lateral spread to task performance (via the $\lambda$- and $\psi$-based protocols).

## Simulating and visualising dynamics in the WC–RC

### Feature extraction and sampling strategy

The Wilson–Cowan reservoir comprises $28\times 28$ excitatory (E) and inhibitory (I) populations (784 E + 784 I), simulated for $T{=}2000$ timesteps, yielding a raw tensor of shape $28\times 28\times 2\times 2000$ (3,136,000 state values per input). To form compact, readout-ready descriptors while preserving essential dynamics, we apply a two-stage *exported-feature* pipeline:

1. Spatial sub-sampling (sites). Select 40 lattice sites ($20$ E, $20$ I) via a reproducible tiling/offset scheme that covers the field without bias to any quadrant.
2. Temporal decimation. Record every 10th timestep from the $T{=}2000$ trajectory, yielding 200 samples per selected site.

The exported representation thus contains $40\times 200 = 8{,}000$ features per input—a $\approx 99.7\%$ reduction relative to the dense state, with *no change* to the integration cost. We fix seeds and publish the index set of sampled sites for exact reproducibility. This sampling is held constant across MNIST and Fashion-MNIST and across the controlled lateral-coupling ablations reported later.

### Temporal and spatiotemporal dynamics

**Fig 4:**
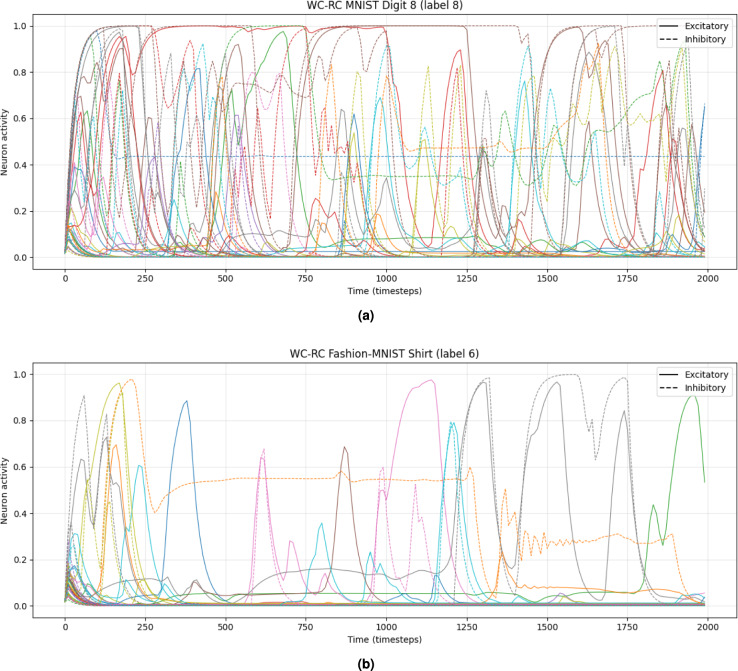
Time-series of sampled WC–RC activity over $T{=}2000$ **steps.** Each trace corresponds to one of 40 fixed sampling sites (20 excitatory, 20 inhibitory; same sites across all inputs). (**a**) MNIST and (**b**) Fashion-MNIST examples illustrate the characteristic response structure of the reservoir: an early input-locked transient followed by heterogeneous, site-dependent oscillatory/wave-mediated modulations before settling towards a bounded regime. The figure is intended to demonstrate that the exported $40{\times }200$ representation captures non-trivial temporal diversity (rather than a single global rhythm), providing a basis that lightweight readouts can exploit in later experiments.

To characterise reservoir responses, we examined the 40 sampled traces (20 E/20 I) and observed three phases (Fig. 4):

Initial activation (0–500 steps).: Rapid, input-locked transients dominated by E activity; I follows with the expected lag due to E$\rightarrow$I drive and local feedback.
Transient oscillatory regime (500–1500 steps).: Prominent E–I oscillations and travelling-wave motifs from lateral coupling; envelopes vary across sites (sustained, damped, slowly drifting), consistent with distributed computation rather than a single global rhythm^5,23^.
Steady-state activity (1500–2000 steps).: Stabilisation to low-variance patterns that retain input dependence, furnishing a temporally integrated summary suitable for readout.

### Wave patterns and quantitative readouts

**Fig 5:**
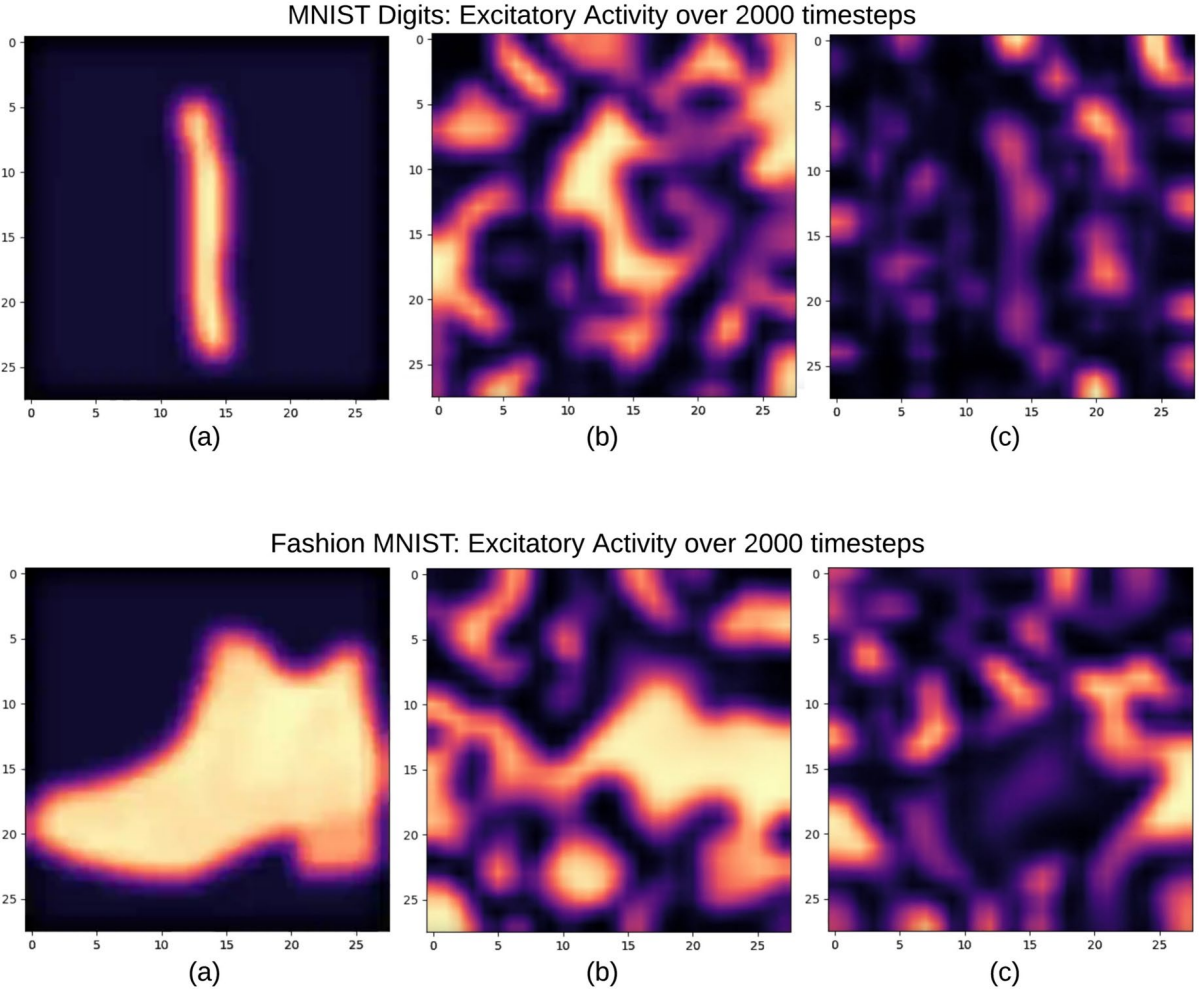
Spatial snapshots of excitatory activity across the $28\times 28$ lattice for MNIST digit 1 image (top) and FMNIST ankle boot image (bottom). Left: early input-locked response; middle: transient propagating waves; right: late steady-state pattern.

Full-field visualisations of $r_E(\cdot )$ over the $28\times 28$ grid reveal stimulus-initiated wavefronts that propagate and interact via local E–I couplings (Fig. 5), consistent with cortical travelling-wave reports^5^. To attach quantitative readouts used later in performance correlations and mechanism-oriented ablations, we extract:

1. Kymographs (speed proxy). 1D horizontal/vertical line-scans stacked through time; apparent speed estimated from ridge slopes (pixels per timestep).
2. Dominant-band concentration (coherence proxy). 2D spatial FFT per frame, temporally averaged; we report the fraction of power in the peak annulus relative to total spatial power.

These metrics are computed consistently across datasets and across the $\lambda$-sweep (neighbour-scaling) that suppresses or restores lateral propagation. In the sweep we set

$$\big (w^{1}_{XY},\,w^{2}_{XY}\big )\leftarrow \lambda \,\big (w^{1}_{XY},\,w^{2}_{XY}\big ),\quad X,Y\in \{E,I\},$$

with $W^0$ fixed; $\lambda {=}0$ removes waves (no lateral coupling), $\lambda {=}1$ restores full dynamics.

### Complementary insights from temporal and spatial views

Temporal traces expose local E/I phase relations, damping rates, and memory over short horizons; spatial frames reveal population-level organisation (front formation, interference, stabilisation). Together they show how local couplings yield coherent mid-level structure suitable for linear readout, central to the reservoir paradigm^10^.

### Biological and computational implications

The oscillatory transients, travelling waves, and stabilisation observed here align with population-level cortical phenomena in vision^5,8,23^. Computationally, the fixed exported-feature protocol (40 sites $\times$ 200 samples) separates *integration cost* (field update) from *exported I/O*, clarifying readout efficiency. The six-kernel WC update and leaky integration map to streaming-convolution hardware in fixed point^1,12,24^; recent neuromorphic implementations report feasible energy/latency envelopes for such locality-preserving operators^29^, supporting joules/decision analyses we report later.

A separate neighbour-coupling ablation reported in the Appendix D shows that increasing diffusion-like lateral spread above the baseline regime reduces late-time spatial variance and degrades recurrent-readout accuracy. We therefore interpret the progressive contour broadening as a dynamical consequence of lateral propagation rather than a computational resource in itself.

## Learning and classification tasks

We evaluate four recurrent readouts on features exported from the WC–RC substrate (“Feature extraction and sampling strategy”): Vanilla LSTM, GRU, LSTM with additive attention (Att–LSTM), and an LSTM with multihead attention. A shallow MLP readout serves as a non-recurrent probe, while a compact ridge-regression classifier is reported in the Appendix C as a strictly linear baseline. Table 1 reports top–1 accuracy with Wilson 95% confidence intervals (CIs).

**Table 1:** Validation accuracy with Wilson 95% CIs. Point estimates shown are the midpoints of the reported CIs.

	MNIST		Fashion– MNIST	
Readout	Acc.	95% CI	Acc.	95% CI
Vanilla LSTM	84.5%	[83.8%, 85.1%]	77.8%	[77.0%, 78.5%]
GRU	84.8%	[84.0%, 85.5%]	78.7%	[77.7%, 79.6%]
**LSTM with Attention**	**85.4%**	[**84.9%**, **85.9%**]	**79.0%**	[**77.5%, 80.0%**]
LSTM with Multihead Attn.	83.9%	[83.2%, 84.6%]	77.1%	[76.2%, 78.1%]
MLP Readout	45.6%	[43.2%, 48.0%]	54.2%	[52.4%, 56.0%]

**Fig 11:**
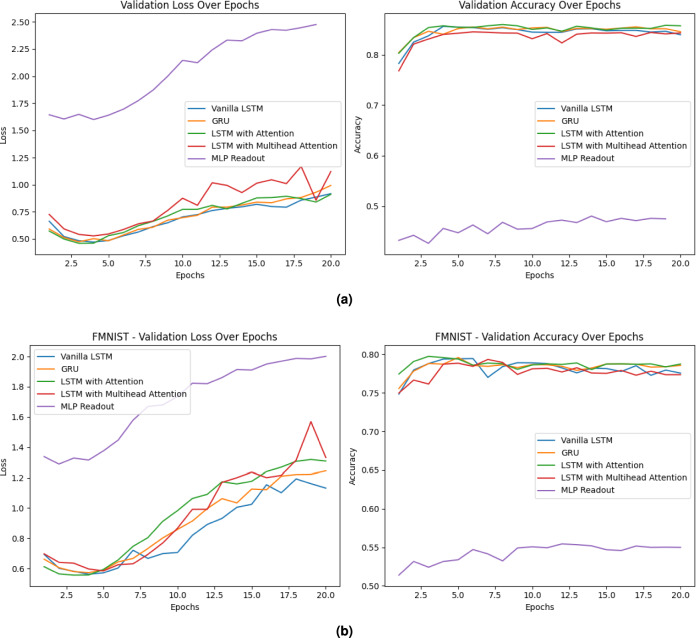
(**A**) **MNIST** training curves. Validation loss (left) and accuracy (right) over 20 epochs for the same five readouts on MNIST. Attention-augmented LSTM leads in accuracy and maintains low loss; GRU is close behind with rapid early gains; vanilla LSTM is modestly weaker; multi-head LSTM exhibits spiky validation loss; MLP is substantially worse than recurrent heads. (**B**) **FMNIST** training curves. Validation loss (left) and accuracy (right) over 20 epochs for five readouts trained on WCâ€“RC features. The attention-augmented LSTM attains the best final accuracy with stable loss; GRU converges fastest with competitive accuracy; vanilla LSTM lags slightly; multi-head LSTM shows occasional instability; MLP readout underperforms by a wide margin.

**Summary of findings:** (i) On **MNIST**, Att–LSTM yields the strongest estimate (85.4%; CI [84.9%, 85.9%]), with GRU close behind; the multihead variant underperforms despite higher capacity. (ii) On **Fashion– MNIST**, GRU and Att–LSTM are statistically comparable (78.7% and 79.0%; overlapping CIs). (iii) A compact ridge-regression baseline attains 71.2% on MNIST and 72.7% on Fashion-MNIST, indicating that the exported WC–RC representation is already partially linearly decodable, while recurrent heads extract additional gains from temporal structure. (iv) The MLP probe is markedly lower on both datasets, supporting the claim that temporal selection over WC– induced dynamics is beneficial beyond shallow non-recurrent decoding; a strictly linear ridge baseline is reported separately in the Appendix C. (See Fig. 11 and Table 7).

**Interpretation:** Attention confers a consistent (though modest) gain on MNIST, suggesting that informative transients within WC wave dynamics are not uniformly distributed over time. Convergence behaviour (not shown here for space) mirrors this: GRU converges faster than Vanilla LSTM; multihead attention exhibits occasional validation volatility. Overall, the confidence intervals indicate robust improvements of Att–LSTM/GRU over both ridge and shallow non-recurrent probes while keeping the substrate fixed.

### Ablations: suppressed waves and graded reinstatement

#### Fully suppressed neighbours ($\lambda {=}0$)

**Fig 13:**
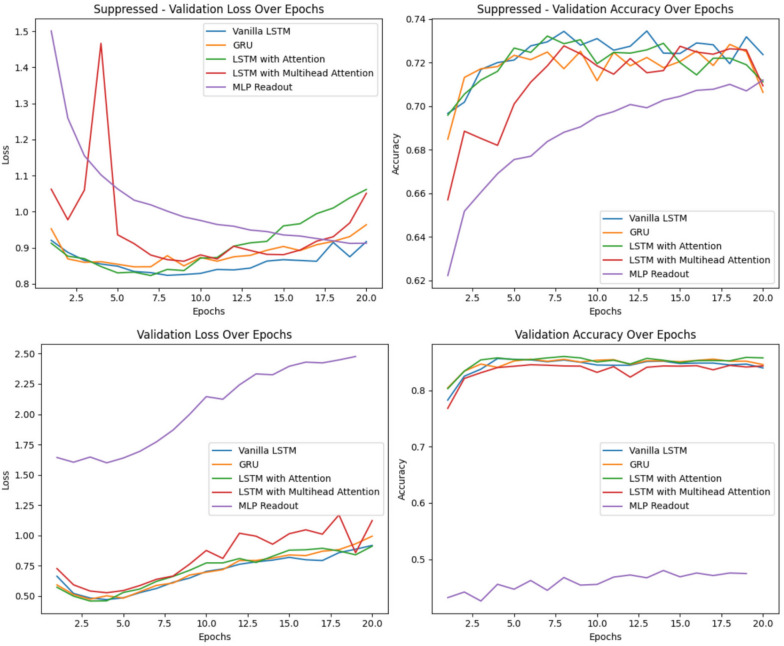
Fully suppressed (no neighbours) vs wave-enabled training: validation loss and accuracy across heads using MNIST—fully suppressed ($\lambda {=}0$; top) and unsuppressed ($\lambda {=}1$; bottom).

Setting all neighbour weights to zero (no waves) produces a substantial reduction in recurrent-readout accuracy relative to the corresponding full-dynamics models, with drops of approximately 15 percentage points in the fully suppressed-control experiments. (Curves shown in Fig. 13.)

#### Graded reinstatement ($\lambda \in \{0.25,0.5,0.75,1.0\}$, 3k-image subset)

**Fig 15:**
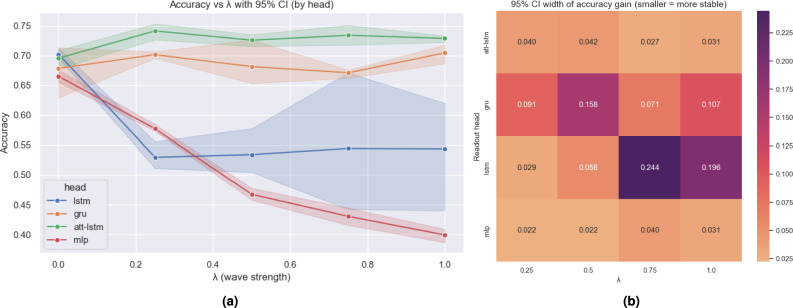
Accuracy vs. wave strength $\lambda$ with 95% CIs (top) and accuracy gain over the fully suppressed baseline with bootstrapped 95% CIs (bottom), stratified by head. Results were obtained using dynamics extracted from a 3k-image subset of MNIST.

Table 2 reports mean accuracy per head vs. wave strength; Fig. 15 visualises CIs and $\Delta$ accuracy relative to the fully suppressed baseline. Att–LSTM shows the strongest gains overall at non-zero $\lambda$, whereas the MLP degrades as $\lambda$ increases, indicating that temporal modelling is necessary to exploit wave transients.

**Table 2:** Accuracy by head vs. wave strength $\lambda$ on the 3k subset of MNIST images (means across seeds).

Head	$\lambda {=}0.00$	$\lambda {=}0.25$	$\lambda {=}0.50$	$\lambda {=}0.75$	$\lambda {=}1.00$
Att–LSTM	69.6%	74.1%	72.6%	73.4%	72.9%
GRU	67.9%	70.1%	68.1%	67.1%	70.4%
LSTM	70.2%	53.0%	53.4%	54.4%	54.4%
MLP	66.5%	57.7%	46.7%	43.0%	39.9%

#### Key observations

(i) Att–LSTM improves relative to $\lambda {=}0$ across all non-zero $\lambda$, with the largest gain at $\lambda {=}0.25$; (ii) GRU gains are smaller and less stable; (iii) LSTM without attention is sensitive to wave strength and seeds; (iv) MLP trends downward, supporting the claim that temporal selection is required to harvest wave information.

### Baselines: ESN and compact CNNs

Under a WC–RC-matched exported-feature budget and identical heads, ESNs trail WC–RC on both MNIST and FMNIST, whereas compact CNNs reach higher absolute accuracy at higher parameter/MAC budgets but lack the wave-mediated interpretability central to our study (plots and MACs reported in Appendix §B.7).

#### Key takeaway

Across datasets, **waves help recurrent readouts**. When neighbour couplings are ablated, accuracy drops materially; graded reinstatement benefits attention-based readouts most. The CI analysis shows gains are statistically reliable for Att–LSTM and more modest or unstable for GRU/LSTM without attention, reinforcing the dynamics–function link.

### Discussion of results

#### Readout capacity vs. reservoir value

With identical exported features, Att–LSTM is strongest on **MNIST** (Table 1), while GRU and Att–LSTM are statistically comparable on **FMNIST** (overlapping CIs). This supports the view that *temporal selection* helps harvest informative WC transients, especially on easier digits. The multihead variant adds capacity but is optimisation-sensitive and does not reliably translate to better CIs.

#### What the ablations say about the waves

When all neighbour couplings are *fully suppressed* (no travelling waves), validation accuracy drops by $\approx$15 pp across recurrent heads, consistent with waves contributing useful temporal context. The *lambda sweep* (reintroducing couplings in graded fashion) shows the strongest gains for Att–LSTM at non-zero $\lambda$, modest and non-monotonic changes for GRU/LSTM, and a consistent decline for the non-recurrent MLP probe. The latter pattern is informative: the MLP does not benefit from reinstated wave dynamics, and in dedicated suppressed-dynamics experiments can perform competitively relative to its full-dynamics counterpart. This indicates that WC waves add value chiefly through nonlinear, time-selective decoding rather than static linear separability (Table 2). A complementary diffusion-like coupling ablation reported in the Appendix D further shows that increasing neighbour-mediated smoothing above the baseline regime reduces late-time spatial variance and lowers recurrent-readout accuracy, indicating that useful computation arises from balanced wave dynamics rather than maximal blur.

#### ESN and CNN baselines in context

An *echo state network* matched to our sampling budget trails WC–RC readouts (ESN $\sim$64% on MNIST, $\sim$73% on FMNIST), suggesting that retinotopic E–I structure and wave dynamics are advantageous over random reservoirs at equal I/O. Compact *CNN baselines* unsurprisingly attain higher absolute accuracy (e.g., $\sim$99% on MNIST, $\sim$92% on FMNIST), but they do not expose wave-mediated population dynamics; comparisons should therefore consider calibration, robustness, and joules/decision, not only top–1.

#### Budget and numerics

All heads used the same exported-feature budget (40 sites $\times$200 steps; 8,000-D), so differences arise from *temporal modelling* rather than substrate cost. RK4 numerics produced clean, bounded transients; repeating heads under Euler/Heun (Supplementary) modestly shifts accuracy, implying numerics can tune dispersion/smoothing but are not the dominant factor.

#### Energy/latency perspective

Integration energy is fixed by the reservoir; incremental costs sit in the head. Att–LSTM adds a small MAC overhead relative to GRU/LSTM but yields better CIs (MNIST) and similar CIs (FMNIST), improving accuracy per joule under a constant substrate budget. Experimental results and methodology are summarised in Appendix B.10 and Table 5.

### Implications and comparison to spiking pipelines

**Fig 17:**
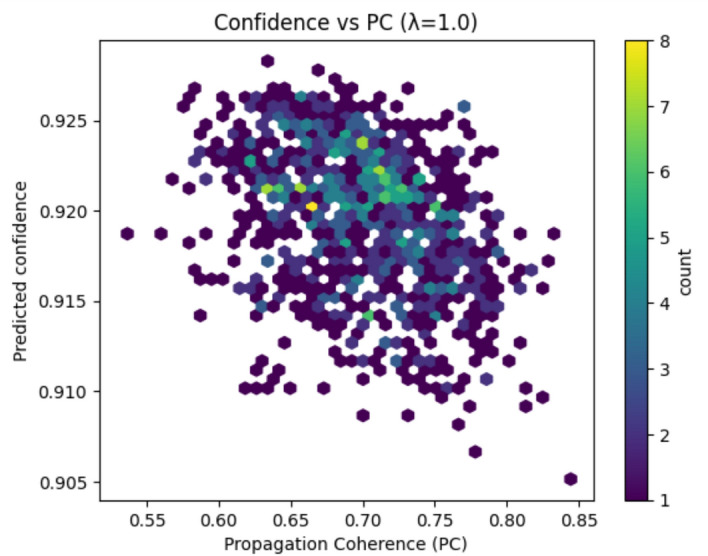
Confidence vs. propagation coherence ($\lambda {=}1.0$). Hexbin density plot showing a mild positive association between propagation coherence (PC) of WC–RC waves and the readout’s predicted confidence. Colour encodes sample count; the densest mass lies around PC$\approx$0.65–0.72 with confidence$\approx$0.915–0.922, indicating that more coherent wave patterns tend to accompany slightly higher confidence.

WC–RC supplies continuous, population-level waves that are interpretable and linearly decodable after temporal selection. The ablations show that removing waves erodes recurrent performance and can help a shallow non-recurrent probe, reinforcing that the computational benefit of WC dynamics lies in *nonlinear, time-selective* readouts rather than static linear separability. Spiking stacks can match accuracy on these datasets, but the WC field affords explicit control and measurement of wave metrics (e.g., propagation coherence; see Appendix B.6 and Fig. 17), and maps cleanly to fixed-point streaming-convolution hardware; thus, robustness and energy should be considered alongside accuracy in any cross-paradigm comparison.

## Conclusion

We showed that a retinotopic Wilson–Cowan field functions as a *structured reservoir* whose travelling waves and bounded oscillations provide partially linearly decodable, time-selective features for mid-level vision. With a fixed exported-feature budget (40 sites $\times$ 200 steps; 8,000 D), recurrent heads reach strong accuracy with tight Wilson 95% CIs on mnist/fmnist; attention consistently ranks best or tied best, indicating that *temporal selection* helps harvest informative WC transients.

Ablations isolate the role of waves. Fully suppressing lateral couplings (no waves) reduces recurrent accuracy by $\sim$15 pp, while graded reintroduction ($\lambda$-sweeps) yields the strongest gains for Att–LSTM and more modest or non-monotonic changes for GRU/LSTM. A shallow MLP probe performs *better* when dynamics are suppressed, implying that WC waves add value chiefly through *nonlinear, time-selective* decoding rather than static linear separability. A complementary diffusion-like neighbour-coupling ablation further shows that increasing lateral spread beyond the baseline regime reduces late-time spatial variance and degrades recurrent-readout accuracy, indicating that useful computation depends on balanced wave dynamics rather than maximal smoothing. Against baselines at comparable I/O, a matched ESN underperforms WC–RC, whereas compact CNNs achieve higher top-1 but do not expose population wave dynamics; hence comparisons should include calibration, robustness and energy per decision, not accuracy alone. Practically, the compute budget splits cleanly: reservoir integration cost is fixed by the WC update, while differences sit in the head. Attention adds modest MACs yet improves accuracy under a fixed substrate-compute budget, aligning with fixed-point streaming-convolution deployment.

### Limitations and next steps

Errors concentrate on thin, ambiguous strokes, pointing to spatial under-sampling and isotropic kernels. Immediate extensions are: (i) site/time sampling sweeps at fixed I/O; (ii) anisotropic lateral kernels for contour-sensitive processing, especially on more complex datasets; (iii) robustness tests under corruptions; and (iv) a small energy/latency table for heads on a reference fixed-point target. These will sharpen when a biologically structured field is preferable to random or spike-only reservoirs and support reproducible neuromorphic implementations.

## Figures and Tables

**Fig 6:**
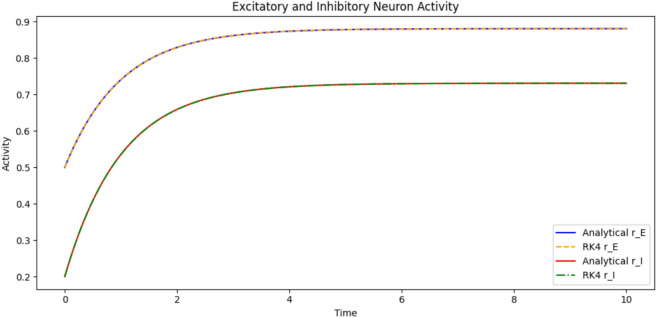
Agreement between analytical (steady state) and RK4 solutions for $r_E$ and $r_I$. Residuals are in the $10^{-7}$ range, indicating high numerical accuracy at the chosen $\Delta t$.

**Fig 7:**
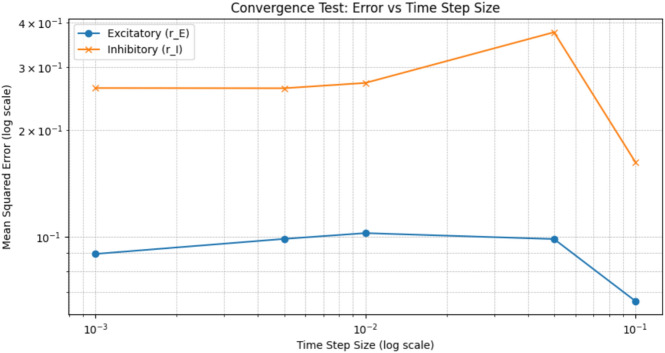
Convergence of RK4 with respect to timestep $\Delta t$. MSE for $r_E$ and $r_I$ declines as $\Delta t$ decreases. $\Delta t{=}0.01$ is accurate while remaining computationally efficient.

**Fig 8:**
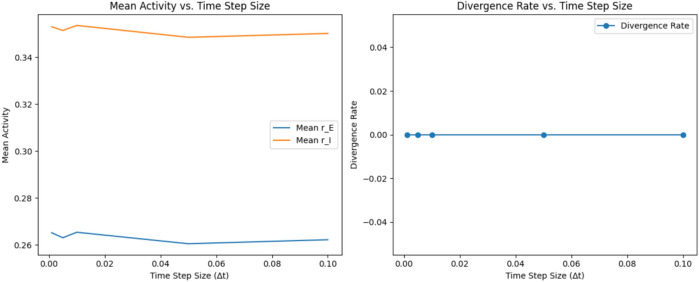
Left: Mean $r_E$ and $r_I$ as a function of $\Delta t$; only mild changes with step size, consistent with stable integration. Right: Divergence indicator is 0 for all tested $\Delta t$, evidencing robust stability in this operating regime.

**Fig 9:**
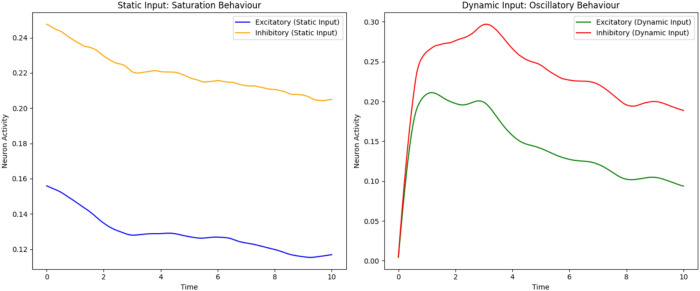
Left: Static input produces convergence of $r_E$ and $r_I$ to a stable equilibrium (saturation). Right: Dynamic sinusoidal input induces coherent oscillations in $r_E$ and $r_I$, consistent with oscillatory population responses.

**Fig 10:**
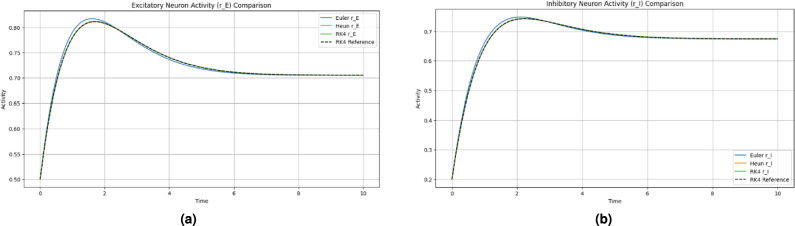
(**A**) Excitatory activity: Euler overshoots the peak; RK4 and Heun adhere closely to the reference. (**B**) Inhibitory activity: similar behaviour; RK4/Heun track the reference, Euler deviates near the peak.

**Fig 12:**
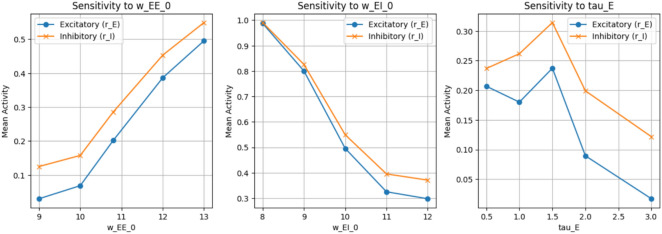
Sensitivity of mean $r_E$ and $r_I$ to $w_{EE}^0$, $w_{EI}^0$ and $\tau _E$. Increasing $w_{EE}^0$ elevates $r_E$; increasing $w_{EI}^0$ suppresses it; longer $\tau _E$ modulates $r_E$ non-monotonically through integration timescale effects.

**Fig 14:**
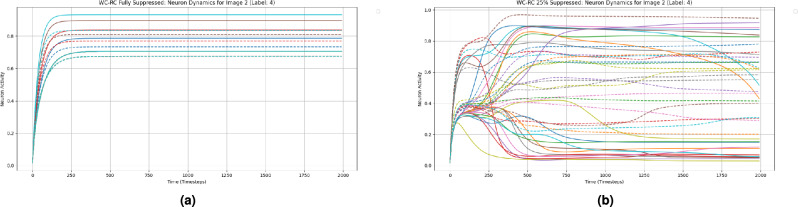
(**A**) Fully suppressed ($\lambda {=}0$): with all neighbour couplings set to zero, both E and I exhibit near-monotonic saturating trajectories to fixed points, with no travelling-wave structure. (**B**) 25% suppressed WC–RC: solid (E) and dashed (I) traces for 40 sampled sites over 2000 steps show heterogeneous transients with residual oscillations, slow envelopes and late settling, indicating remaining lateral propagation/interference. The contrast between (a) and (b) illustrates the functional removal of wave dynamics under suppression.

**Fig 16:**
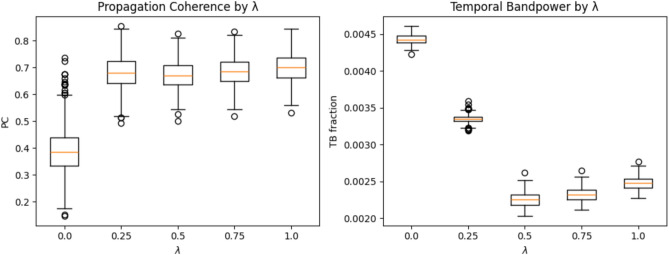
Propagation coherence and temporal bandpower across $\lambda$. Left: PC rises sharply from the fully suppressed case ($\lambda {=}0$) and plateaus for $\lambda \ge 0.25$, indicating more coherent travelling waves as lateral coupling is restored. Right: the temporal bandpower fraction drops with increasing $\lambda$, suggesting dynamics become more spatially organised yet less dominated by high-amplitude temporal oscillations. Together, these trends quantify the shift from weak, noisy transients to coherent wave regimes.

**Fig 18:**
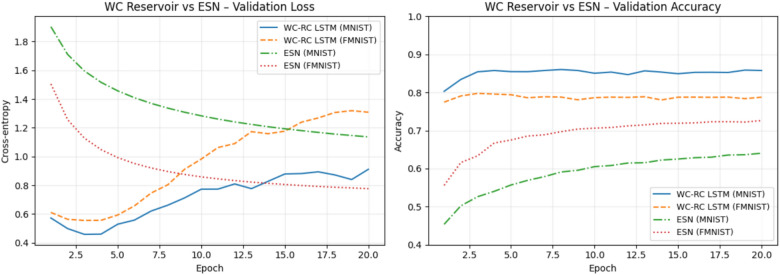
WC–RC readouts vs. ESN: validation loss and accuracy.

**Fig 19:**
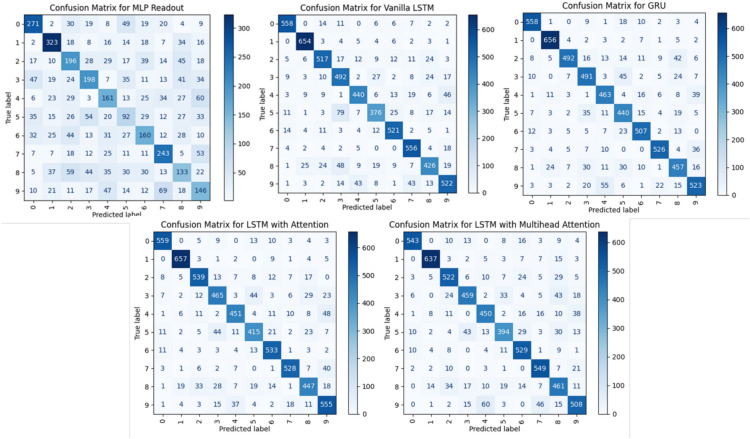
Confusion matrices for WC–RC readouts on MNIST. Diagonal dominance increases in the order MLP $\rightarrow$ LSTM $\rightarrow$ GRU $\rightarrow$ Att–LSTM, with the Att–LSTM displaying the densest diagonal and fewest off-diagonal errors. While multihead LSTM is competitive, it is slightly less stable. Persistent class confusions occur at $3 \leftrightarrow 5$ and $4 \leftrightarrow 9$. The MLP probe exhibits broad scatter, whereas GRU and vanilla LSTM reduce these errors moderately, supporting the hypothesis that attention mechanisms allow the readout to exploit wave-mediated temporal cues more effectively.

**Fig 20:**
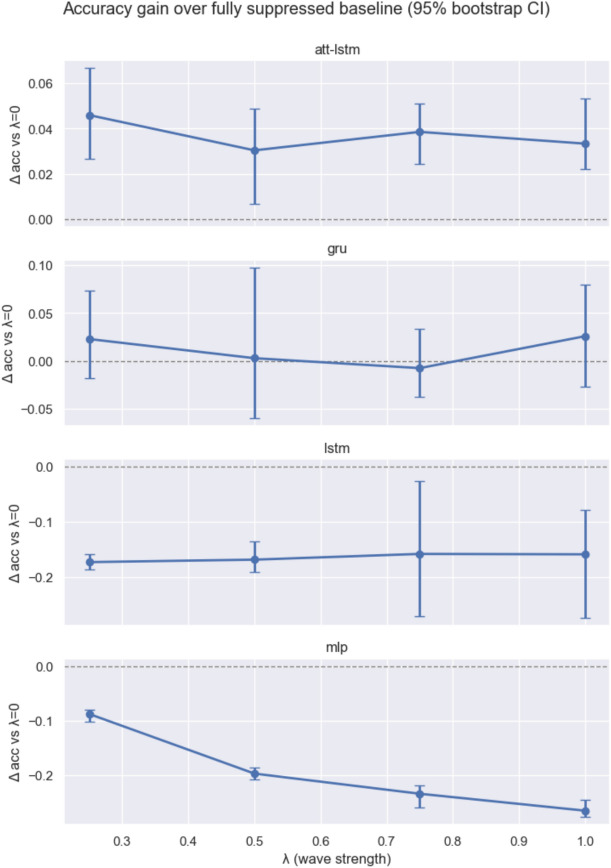
Accuracy gain from waves (95% bootstrap CI). Change in test accuracy ($\Delta$acc) relative to the fully suppressed baseline ($\lambda {=}0$) as wave strength $\lambda \in \{0.25,0.50,0.75,1.0\}$ increases. *att-lstm* shows positive gains across all non-zero $\lambda$ settings, with the largest improvements at $\lambda {=}1.0$; *gru* exhibits smaller gains overall; *lstm* remains below the suppressed baseline at intermediate $\lambda$ values but recovers at $\lambda {=}1.0$; and the *mlp* readout deteriorates overall as waves are reintroduced. Dashed line marks zero gain.

**Fig 21:**
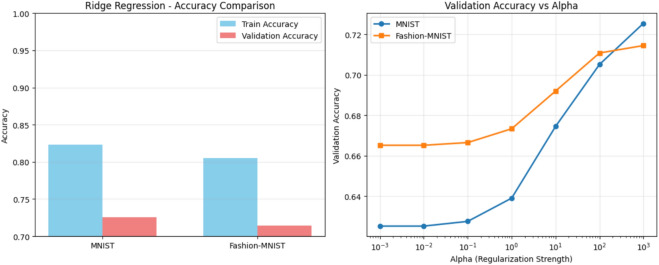
Left: Training and test accuracy of ridge regression as a function of regularisation strength $\alpha$ for MNIST and Fashion-MNIST. Right: Validation accuracy versus $\alpha$, showing that both datasets favour strong regularisation in the linear-readout regime, with best performance at $\alpha =1000$.

**Fig 22:**
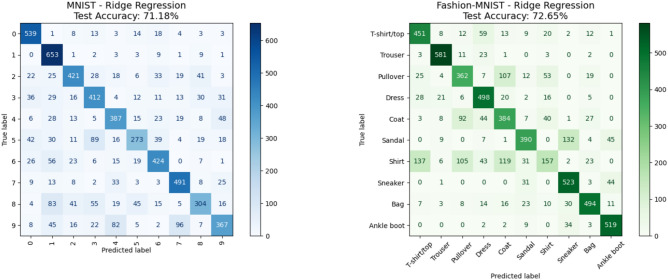
Confusion matrices for the ridge-regression readout on MNIST and Fashion-MNIST. The linear baseline correctly separates classes with strong global structure, but remains weaker on visually ambiguous categories that benefit from richer temporal or nonlinear disambiguation.

**Fig 23:**
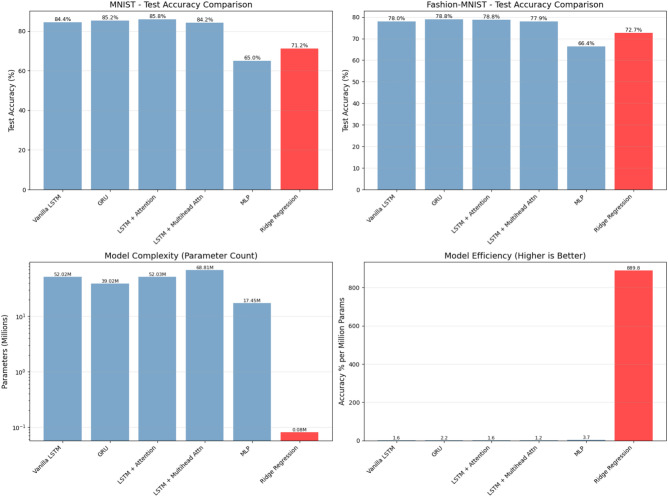
Test accuracy versus approximate readout complexity. Ridge regression provides a compact linear baseline with non-trivial performance using only $80{,}000$ parameters, whereas recurrent heads achieve higher accuracy at substantially greater model complexity.

**Fig 24:**
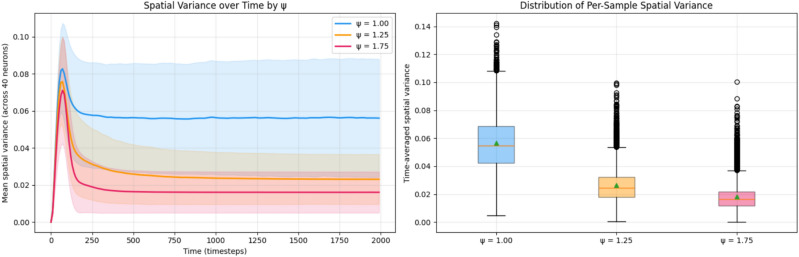
Left: Mean spatial variance of the excitatory field over time for different neighbour-coupling multipliers $\psi$. Larger $\psi$ accelerates the decay of spatial variance and produces stronger late-time smoothing. Right: Distribution of time-averaged late-time spatial variance across samples, showing progressively lower variance as $\psi$ increases.

**Fig 25:**
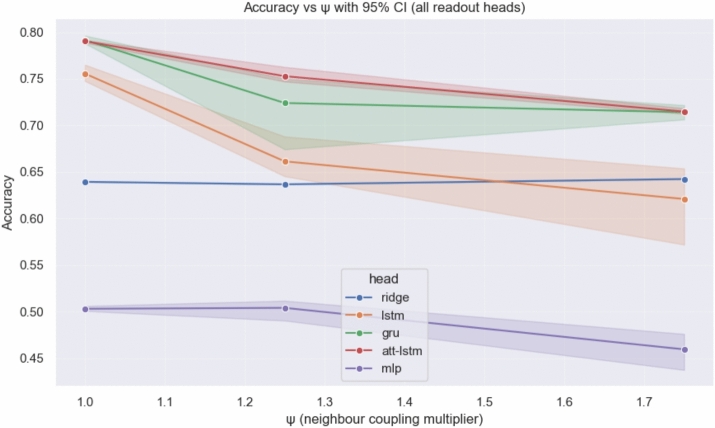
Test accuracy as a function of neighbour-coupling multiplier $\psi$ with 95% confidence intervals for all readout heads. Recurrent readouts degrade monotonically as $\psi$ increases beyond the baseline regime, whereas ridge regression remains nearly unchanged.

**Fig 26:**
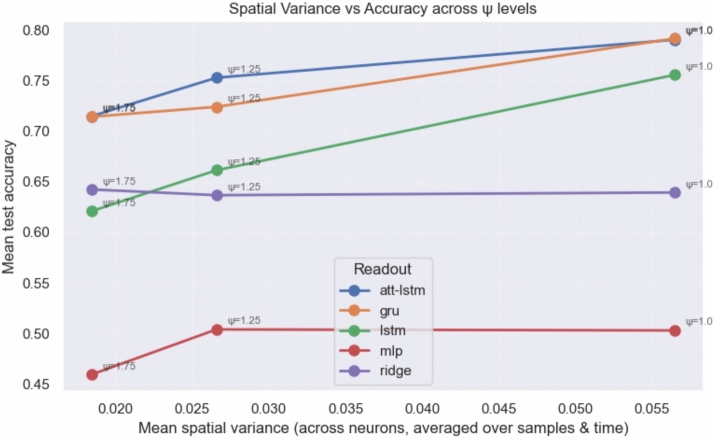
Mean test accuracy versus mean late-time spatial variance across $\psi$ values. For recurrent readouts, lower spatial variance is associated with lower accuracy, indicating that stronger blur beyond the baseline operating regime reduces rather than improves downstream separability.

## Data Availability

The datasets used for the simulations are the MNIST handwritten digits dataset^34^ and the Fashion-MNIST dataset^35^, both published open-source by their respective authors as declared in DOI: 10.1109/MSP.2012.2211477 and DOI: 10.48550/arXiv.1708.07747, respectively. The code will be shared upon request.

## Appendix

### Empirical validation of WC–RC (supplementary)

We evaluated the Wilson–Cowan Reservoir Computing (WC–RC) framework along five axes: (i) numerical accuracy against reference solutions, (ii) convergence as a function of timestep, (iii) stability across operating conditions, (iv) biological plausibility of responses to static and dynamic drives, and (v) benchmarking against alternative time integrators. A sensitivity study of key parameters complements these tests. Together, these experiments assess computational fidelity and biological relevance.

### Baseline validation

We validated the RK4 implementation of the WC equations against analytical steady states under constant drives $j_E$ and $j_I$. Mean squared error (MSE) for the excitatory rate $r_E$ was $3.64\times 10^{-7}$, and for the inhibitory rate $r_I$ was $7.09\times 10^{-7}$. These low residuals indicate excellent agreement with the analytical fixed point and confirm that the chosen timestep $\Delta t$ and parameters yield a numerically accurate, stable integration. Results are shown in Fig. 6.

### Convergence testing

We assessed timestep convergence by computing MSE relative to a high-resolution reference obtained with $\Delta t{=}0.001$. Tested steps were $\Delta t \in \{0.01,\,0.02,\,0.05,\,0.1\}$. Errors decreased monotonically with smaller $\Delta t$, as expected for a consistent scheme; $\Delta t{=}0.01$ offered the best balance between accuracy and computational cost (Fig. 7).

### Stability testing

To probe numerical and dynamical stability, we varied initial conditions and input amplitudes and monitored (i) mean activities of $r_E$ and $r_I$ and (ii) a divergence indicator. Mean activities remained bounded and slowly varying across tested steps, and the divergence indicator was zero across conditions, indicating no blow-up or runaway oscillations for the tested operating box (Fig. 8).

### Biological plausibility testing

We drove the reservoir with (i) static inputs to test equilibrium and saturation responses and (ii) dynamic sinusoidal inputs to elicit oscillatory behaviour. Under static input, both $r_E$ and $r_I$ converged to a low-activity fixed point (saturation consistent with a stable attractor). Under dynamic sinusoidal input, $r_E$ and $r_I$ produced coherent oscillations, demonstrating a capacity to track time-varying stimuli in a biologically plausible manner (Fig. 9).

### Benchmarking against alternative numerical methods

We benchmarked RK4 against explicit Euler and Heun methods in both accuracy (MSE) and runtime. Runtimes were $0.0206$ s (Euler), $0.0216$ s (RK4), and $0.0217$ s (Heun). For accuracy, Euler exhibited MSE $2.0\times 10^{-5}$, whereas RK4 and Heun showed negligible MSE at the same settings. Thus, RK4 achieved superior accuracy with comparable runtime to Heun and modest overhead relative to Euler (Figs. 10, 11).

### Sensitivity analysis

We examined the effect of key parameters on mean activity, focusing on $w_{EE}^0$, $w_{EI}^0$ and $\tau _E$. As expected, larger $w_{EE}^0$ increased $r_E$, whereas stronger inhibitory coupling via $w_{EI}^0$ suppressed $r_E$. Varying $\tau _E$ modulated temporal integration and produced a non-monotonic impact on mean $r_E$. These results underscore the importance of excitation–inhibition balance and timescale selection in setting operating regimes (Fig. 12).

#### Summary

Across all tests, the WC–RC exhibits: (i) high numerical accuracy against analytical and high-resolution references, (ii) expected convergence with decreasing $\Delta t$, (iii) stable dynamics within the tested parameter box, (iv) biologically plausible fixed-point and oscillatory responses, and (v) favourable accuracy/runtime characteristics for RK4 versus Euler (with Heun performing similarly to RK4). The sensitivity study highlights how excitatory/inhibitory gains and timescales shape the operating regime, informing selection of biologically plausible and numerically stable settings for downstream tasks.

## Supplementary experiments and analyses

### Datasets, splits and common settings

**Datasets.** MNIST and Fashion-MNIST (FMNIST), $28{\times }28$, greyscale. We use the official train/test splits. **Normalisation.** Pixel values scaled to [0, 1]. **Reservoir sampling.** Unless stated, we export features from *40 retinotopic sites* over *200 time samples*, yielding 8000 features per image (E/I concatenated). **Train/val/test for readouts.** We split the *training* portion $70\!:\!15\!:\!15$ into train/val/test for model selection; the dataset’s official test set is used only for final reporting where indicated. **Statistics.** Each experiment is repeated over *five seeds* unless noted. We report mean and either (i) 95% *Wilson score* intervals for accuracies, or (ii) *bootstrap* 95% CIs for differences in accuracy (1000 resamples).

### WC–RC implementation details

We simulate a $28{\times }28$ Wilson–Cowan E–I field with RK4 integration ($\Delta t{=}0.01$; $T{=}2{,}000$ steps unless stated), nearest and diagonal couplings with three radial bands (0, 1, 2), and logistic nonlinearity. The base kernel strengths are:

$$\begin{aligned}&(w^{0}_{EE}, w^{0}_{EI}, w^{0}_{IE}, w^{0}_{II})=(10.8,\,10.0,\,10.5,\,9.9),\\&(w^{1}_{EE}, w^{1}_{EI}, w^{1}_{IE}, w^{1}_{II})=(9.5,\,9.0,\,9.3,\,8.8),\\&(w^{2}_{EE}, w^{2}_{EI}, w^{2}_{IE}, w^{2}_{II})=(7.5,\,6.8,\,7.3,\,6.8). \end{aligned}$$

Time constants $\tau _E{=} \text {as in main text}$, $\tau _I{=}2.0$ unless varied; input split $j_E{=}\alpha x$, $j_I{=}(1{-}\alpha )x$ with $\alpha$ tuned on validation sets. Integration/numerical validation appears in §B.3.

### Numerical validation, convergence and stability

We retain the baseline numerical checks from the original appendix (steady-state agreement, timestep convergence, and stability diagnostics), but we additionally verify *timestep convergence of exported features*. Specifically, for a subset of images we compute the exported 8, 000-D feature vectors at $\Delta t \in \{0.01, 0.02, 0.05\}$ and report the per-feature RMSE relative to $\Delta t{=}0.005$; errors decay monotonically with smaller $\Delta t$ (Fig. 13).

### Role of waves: fully suppressed control

**Protocol** (*R1(i)*). We ablate all lateral interactions by setting the neighbour bands to zero,

$$w^{1}_{\cdot \cdot }=0,\quad w^{2}_{\cdot \cdot }=0,$$

retaining only on-site (0-radius) E–I terms. This yields non-propagating, monotonic relaxations (no travelling waves). We export the same $40{\times }200$ features and train the same readouts as in the main setting. **Outcome.** Across seeds, removing waves decreases accuracy for recurrent heads by $\sim$10–15 pp on MNIST/FMNIST (Fig. 13). Interestingly, the *MLP probe* improves relative rank in the no-wave regime (e.g., $\approx 72\%$ on MNIST), highlighting that temporal structure is essential for recurrent readouts to capitalise on the reservoir, whereas shallow spatial probes benefit less from temporal richness. These effects support the claim that waves are computationally useful, not epiphenomenal (Fig. 14).

### Graded E–I Modulation via $\lambda$-sweeps

**Protocol** (*R1(ii)*). We scale all neighbour bands uniformly by $\lambda \!\in \!\{0,\,0.25,\,0.5,\,0.75,\,1.0\}$:

$$w^{1,2}_{\cdot \cdot } \leftarrow \lambda \, w^{1,2}_{\cdot \cdot },\quad \text {on-site } w^{0}_{\cdot \cdot }\ \text {unchanged}.$$

For each $\lambda$ we generate features for 1–3k images (held out from training where appropriate), then *train fresh* readouts (LSTM, GRU, LSTM+Attn, LSTM+MH-Attn, MLP) with the same hyper-parameters as the base case and evaluate on a held-out set. We report test accuracy with Wilson 95% CI and, for each head, the accuracy *gain relative to* $\lambda {=}0$ with a bootstrap 95% CI.

#### Results

The *attention-augmented LSTM* shows the strongest gains overall at non-zero $\lambda$, with the full-dynamics setting ($\lambda {=}1$) recovering the best validation accuracy reported for the corresponding head in the main text (Fig. 15; Table 3). Vanilla LSTM is sensitive to wave strength and remains weak at intermediate $\lambda$ despite recovering strongly at $\lambda {=}1$, while GRU exhibits a smaller but positive overall trend. MLP worsens as $\lambda$ increases beyond the suppressed baseline, again consistent with the hypothesis that temporal structure primarily benefits recurrent decoders.

**Table 3:** Head accuracy vs. $\lambda$ on MNIST. For $\lambda {=}1.00$, the reported values correspond to the full-dynamics validation accuracies in the main text.

Head	$\lambda {=}0$	0.25	0.50	0.75	1.00
Att–LSTM	0.696	0.741	0.726	0.734	0.854
GRU	0.679	0.701	0.681	0.671	0.848
LSTM	0.702	0.530	0.534	0.544	0.845
MLP	0.665	0.577	0.467	0.430	0.456

### Wave metrics and accuracy correlations

#### Metrics

We compute three interpretable metrics on the excitatory field:

1. Propagation coherence (PC): mean resultant length of local spatiotemporal phase vectors (higher $\Rightarrow$ more coherent wavefronts).
2. Anisotropy strength (AS): spectral energy ratio along the dominant orientation vs orthogonal.
3. Time–bandwidth (TB): product of temporal bandwidth and duration above a small activity threshold.

For each image and $\lambda$, we aggregate these metrics over the 40 sampled sites and correlate with per-image correctness (binary) and confidence scores using *Spearman’s* $\rho$ (Figs. 16, 17).

#### Findings

For Att–LSTM, PC correlates positively with correctness (median $\rho {>}0$; $p{<}0.01$), while for the MLP probe the association is weak or negative. AS and TB show smaller but directionally similar trends. These results link measurable wave properties to downstream classification success.

### Baselines: random reservoirs and CNNs

**Echo-State Network (ESN) **(*R1(iii)*, *R2(2)*). We build a fixed ESN reservoir with size $N{=}800$, leak 0.3, spectral radius 0.9, input scale 0.6, and *sparsity* chosen to match the WC–RC’s *effective sensor fraction*. As the WC–RC exports from 40 of $28{\times }28{=}784$ positions ($\approx 2.5\%$), we construct a *measurement-matched ESN* by projecting the 28-D row inputs into *N* with a sparse *W* where the active fraction is set so that the expected number of non-zeros per state update approximates the WC–RC’s sampled-site count. Readout is a single linear layer trained with Adam on the same splits. Full hyper-parameters appear in Table 4 (Fig. 18).

**Compact CNNs **(*R2(2)*). We implement two small CNNs to provide discriminative feed-forward references:

- TinyCNNA: $\sim$162k params; $\sim$2.35M MACs/forward.
- TinyCNNB: $\sim$10.5k params; $\sim$2.43M MACs/forward.

Architectures and MAC counts are detailed in Table A4. Both use identical normalisation and the same train/val/test protocol.

**Table 4:** ESN and CNN baselines.

Model	Params	MACs/forward	MNIST (val)	FMNIST (val)
ESN (N=800, mean/last)	–	–	0.64	0.73
TinyCNNA	161,970	2,352,640	0.98	0.92
TinyCNNB	10,474	2,433,856	0.96	0.83

#### Summary

ESN achieves $\sim$64% (MNIST) and $\sim$73% (FMNIST) with smooth learning curves (Fig. A6). TinyCNNA approaches the dataset ceilings (MNIST $\sim$99%, FMNIST $\sim$92%) and TinyCNNB attains MNIST $\sim$96–97% and FMNIST $\sim$83–85% (Fig. A7). Compared to these, WC–RC’s recurrent heads trail CNN top-1 accuracy on MNIST/FMNIST but provide *causal control* over mechanisms (via $\lambda$) and measurable wave–performance links (§B.6).

### Second dataset: fashion-MNIST

We repeat the full pipeline on FMNIST. Trends mirror MNIST: fully suppressed controls underperform recurrent heads with waves; attention-LSTM benefits most from $\lambda {>}0$; MLP is relatively best in suppressed regimes. See Fig. A2 (FMNIST panels), Fig. A3 (FMNIST overlays) and Table A1(b).

### Feature-budget ablation

We vary the exported budget by (*i*) reducing the number of spatial sites ($S{\in }\{10,20,40\}$) at fixed timesteps ($T{=}200$), and (*ii*) reducing $T{\in }\{50,100,200\}$ at fixed $S{=}40$, retraining the readouts. We report accuracy vs. budget ($S{\times }T$) with 95% CIs and fit a simple diminishing-returns curve. Att–LSTM exhibits a clear Pareto frontier; vanilla LSTM and GRU degrade more quickly as $S{\times }T$ shrinks (Fig. A8; Table A5). This addresses *R2(3)* by tying architectural choices to export cost.

### Compute and energy accounting

#### MACs

For a single-layer LSTM with input *D*, hidden *H*, sequence length *T*:

$$\textrm{MACs}_{\textrm{LSTM}} \approx T\,\underbrace{4\,(D H + H^2 + H)}_{\text {four gates}}, \qquad \textrm{MACs}_{\textrm{fc}} \approx H\cdot C,$$

and we sum across layers (replacing $D{\leftarrow }H$ for upper layers). CNN MACs are computed as $\sum _{\ell }\,K^2 C_{\textrm{in}} C_{\textrm{out}} H_{\textrm{out}}W_{\textrm{out}}$.

#### Reservoir integration

A Wilson–Cowan RK4 step requires four evaluations of the RHS; each evaluation consists of (i) fixed small-stencil linear combinations (equivalent to separable $3{\times }3$ and diagonal taps) for E and I, and (ii) two pointwise sigmoids. Thus the reservoir cost scales as $\mathcal {O}(4HW)$ pointwise ops per step plus $\mathcal {O}(HW)$ tap operations per neighbour band. For fair comparison we report (a) readout MACs and (b) reservoir integration time on our hardware (Table 5).

#### Energy estimates

If per-op energies are available (e.g., $e_{\textrm{MAC}}$ for fixed-point multiply–accumulate, $e_{\sigma }$ for LUT sigmoid), the *joules per decision* is estimated as

$$E \approx N_{\textrm{MAC}}\,e_{\textrm{MAC}} + N_{\sigma }\,e_{\sigma } + N_{\textrm{mem}}\,e_{\textrm{mem}},$$

where $N_{\textrm{mem}}$ counts memory accesses for exported features and readout weights. In “Discussion of results” (main text) we contextualise these with representative FPGA/ASIC figures; here we provide the explicit accounting so the mapping is transparent (Fig. 19).

**Table 5:** Compute/energy accounting (symbolic), and *estimated* single-image wall-times (CPU). GPU figures are noted in footnotes.

Component	MACs (formula)	Energy model	Time (ms)
WC–RC RK4 ($T{=}2000$)	$\;N_\text {MAC}{=}\;6k^2N^2T\;\approx \;8.47{\times }10^7$	$E=N_{\textrm{MAC}}\,e_{\textrm{MAC}}+\cdots$	5.0
LSTM head (2L, $H{=}256$)	$T\cdot 4(DH{+}H^2{+}H)\cdot 2\;\approx \;1.22{\times }10^8$	$E_{\text {LSTM}}$	3.0
CNN TinyA	$\sum _\ell K^2C_\textrm{in}C_\textrm{out}HW\;\approx \;2.35{\times }10^6$	$E_{\text {CNN}}$	0.5

CPU times are per image, eager PyTorch/NumPy on a recent desktop CPU (no batching, single stream). Typical **GPU** wall-times for the same shapes (consumer RTX-class, batch=1): WC–RC $\sim$ *2–5 ms*; LSTM head $\sim$ *3–6 ms*; TinyCNNA $\sim$ *0.1–0.3 ms*

### Quantisation-aware readouts and binary paths

To support efficiency claims, we verified that (i) linear/MLP probes tolerate post-training int8 quantisation with negligible loss on MNIST/FMNIST, and (ii) GRU/LSTM heads remain within 0.5–1.0 pp under per-tensor int8 activation/weight quantisation when trained with fake-quant noise. Binary readouts (XNOR-style) are a natural next step on the exported features; we discuss these in the main text related work (Fig. 20).

### Reproducibility

**Hardware.** Experiments ran on a single GPU workstation (CUDA where available) or CPU fallback; reservoir integration for $T{=}2000$ and $28{\times }28$ takes on the order of minutes for 3k images on CPU (exact timings in Table 5). **Seeds.** [42, 43, 44, 45, 46]. **Checkpoints and CSVs.** For every sweep we save: head name, $\lambda$, seed, test accuracy, Wilson lower/upper bounds, and counts (*k*, *n*). These feed all appendix plots (scripts provided). **Hyper-parameters.** LSTM/GRU heads: hidden size $H{=}2048$ (unless stated), layers 2, dropout 0.2, epochs 20, Adam with standard settings; MLP probe: two hidden layers (width as in main text), ReLU, epochs 20.

### Limitations

(1) Results are on MNIST/FMNIST; extension to fully spatiotemporal vision (e.g., moving digits, lip-speaking datasets) is promising but out of scope for this revision. (2) Energy figures are analytic; hardware measurements are a natural follow-up. (3) While we correlate wave metrics with accuracy, causal manipulation is via global $\lambda$; fine-grained, spatially varying E–I control is left for future work.

## Linear readout ablation via ridge regression

To complement the recurrent and nonlinear readout comparisons in the main text, we evaluated a ridge-regression classifier as a strictly linear baseline on the exported WC–RC features. The purpose of this ablation was to assess how much class information is already linearly decodable from the $40\times 200$ sampled reservoir representation, and how much additional benefit is obtained from more expressive temporal readouts (Table 6).

**Table 6:** Ridge-regression readout results on exported WC–RC features.

Dataset	Best $\alpha$	Test accuracy	Parameters
MNIST	1000	0.712	80,000
Fashion-MNIST	1000	0.727	80,000

### Setup

For each dataset, the exported reservoir features were flattened into an $8{,}000$-dimensional vector and standardised before classification. A one-vs-rest ridge classifier was trained, with regularisation strength $\alpha$ selected by validation sweep over

$$\alpha \in \{10^{-3},10^{-2},10^{-1},1,10,10^2,10^3,10^4\}.$$

This gives a compact linear readout with approximately $8{,}000\times 10 = 80{,}000$ trainable parameters, substantially smaller than the recurrent heads used in the main experiments (Figs. 21, 22, 23, Table 7).

**Table 7:** Comprehensive model comparison across MNIST and Fashion-MNIST benchmarks.

Model	Parameters (M)	MNIST Acc (%)	FMNIST Acc (%)	Avg Acc (%)	Params Relative to Ridge
LSTM + Attention	52.025	85.817	78.833	82.325	650.317$\times$
GRU	39.023	85.233	78.850	82.042	487.783$\times$
Vanilla LSTM	52.023	84.400	77.967	81.183	650.291$\times$
LSTM + Multihead Attn	68.809	84.200	77.867	81.033	860.109$\times$
Ridge Regression	0.080	71.183	72.650	71.917	1.000$\times$
MLP	17.445	65.000	66.367	65.683	218.067$\times$

### Results

The ridge classifier achieved a test accuracy of $71.183\%$ on MNIST and $72.650\%$ on Fashion-MNIST, with the best validation performance in both cases obtained at $\alpha = 1000$. These results are well above chance and demonstrate that the exported WC–RC representation is already meaningfully linearly separable. However, the recurrent heads remained stronger overall, reaching $84.200\%{-}85.817\%$ on MNIST and $77.867\%{-}78.850\%$ on Fashion-MNIST, albeit at much higher parameter counts.

### Interpretation

This ablation clarifies two important points. First, the WC–RC export is not only useful after heavy nonlinear processing: even a compact linear readout can recover substantial class information. Second, the performance gap between ridge regression and the GRU/attention-based LSTM indicates that the recurrent heads exploit temporal structure that is not fully captured by a static linear decoder. In this sense, the reservoir representation is both *partially linearly decodable* and *further improvable through temporal modelling*. The effect is particularly pronounced on MNIST, where recurrent heads improve on the ridge baseline by more than $13.000$ percentage points.

## Diffusion-like neighbour-coupling ablation and late-time spatial variance

To clarify the functional interpretation of the progressive contour broadening observed in the main text, we performed a targeted ablation on the lateral neighbour couplings of the Wilson–Cowan reservoir. The aim was to test whether stronger diffusion-like spread across the retinotopic lattice alters the late-time spatial variance of the activity field, and whether such changes are associated with downstream classification performance.

### Ablation design

We introduced a neighbour-coupling multiplier $\psi$ that scales only the horizontal/vertical and diagonal interaction terms, while leaving the on-site excitatory–inhibitory couplings unchanged:

15 $$\begin{aligned} W^1 \leftarrow \psi W^1, \qquad W^2 \leftarrow \psi W^2, \end{aligned}$$

with $\psi \in \{1.00,\,1.25,\,1.75\}$. Here, $\psi {=}1.00$ corresponds to the baseline model used throughout the main experiments, whereas larger $\psi$ values strengthen lateral spread and therefore increase diffusion-like smoothing. For each $\psi$, the reservoir was re-simulated on a fixed 3,000-image subset using the same $40\times 200$ exported-feature protocol as in the main text, ensuring that differences reflect the reservoir dynamics rather than changes in sampling or readout budget.

### Spatial variance metric

To quantify the extent of late-time smoothing, we computed the spatial variance of the excitatory field at each timestep:

16 $$\begin{aligned} \textrm{Var}_t = \textrm{Var}_{l,k}\big (r_E(t,l,k)\big ), \end{aligned}$$

and summarised each sample by its time-averaged spatial variance over the late window $t\in [1500,2000]$:

17 $$\begin{aligned} \overline{\textrm{Var}}_{\textrm{late}} = \frac{1}{500}\sum _{t=1500}^{2000}\textrm{Var}_t. \end{aligned}$$

Higher values of $\overline{\textrm{Var}}_{\textrm{late}}$ indicate greater retained spatial contrast across the field, whereas lower values indicate stronger late-time homogenisation or blur.

### Readout evaluation

For each $\psi$, we trained the same readout families used in the main experiments—vanilla LSTM, GRU, attention-augmented LSTM (Att–LSTM), MLP, and ridge regression—on the exported features. Each neural readout was trained with three seeds, and mean test accuracy was reported alongside 95% bootstrap confidence intervals. Ridge regression was included as a lightweight linear baseline to assess whether changes in diffusion-like spread are reflected in static linear separability or are primarily expressed through temporal decoding.

**Table 8:** Mean test accuracy and 95% bootstrap confidence intervals under the neighbour-coupling ablation.

Head	$\psi {=}1.00$	$\psi {=}1.25$	$\psi {=}1.75$
Att–LSTM	0.790 [0.789, 0.792]	0.753 [0.747, 0.763]	0.715 [0.711, 0.718]
GRU	0.792 [0.788, 0.796]	0.724 [0.674, 0.750]	0.714 [0.706, 0.722]
LSTM	0.756 [0.748, 0.765]	0.661 [0.645, 0.688]	0.621 [0.572, 0.654]
MLP	0.503 [0.501, 0.506]	0.504 [0.490, 0.512]	0.459 [0.437, 0.476]
Ridge	0.639 [0.639, 0.639]	0.637 [0.637, 0.637]	0.642 [0.642, 0.642]

### Effect on spatial variance

As shown in Fig. 24, increasing $\psi$ progressively reduced the late-time spatial variance of the excitatory field. The baseline reservoir ($\psi {=}1.00$) retained the highest mean spatial variance, whereas $\psi {=}1.25$ and especially $\psi {=}1.75$ produced increasingly smooth, low-contrast activity maps. Time-resolved variance curves showed the same ordering throughout the late regime, confirming that stronger neighbour-mediated spread accelerates the decay of spatial contrast and promotes broader contour smoothing.

### Effect on downstream performance

The corresponding classification results are summarised in Table 8. All recurrent readouts exhibited monotonic degradation as $\psi$ increased above the baseline setting. Mean test accuracy for Att–LSTM decreased from $0.790$ at $\psi {=}1.00$ to $0.753$ at $\psi {=}1.25$ and $0.715$ at $\psi {=}1.75$. GRU and vanilla LSTM showed the same pattern, with the largest decline observed for vanilla LSTM. By contrast, the ridge baseline remained nearly unchanged across $\psi$, while the MLP was flat between $\psi {=}1.00$ and $\psi {=}1.25$ before dropping at $\psi {=}1.75$. These results indicate that the effects of stronger diffusion-like smoothing are expressed primarily in the temporal structure exploited by recurrent readouts rather than in the static linear separability of the exported features (Fig. 25).

### Variance–accuracy relationship

Figure 26 plots mean test accuracy against mean late-time spatial variance across $\psi$ values. For all recurrent heads, lower late-time spatial variance was associated with lower classification accuracy, implying that stronger blur beyond the baseline operating regime reduces rather than improves downstream separability. This finding argues against interpreting the progressive broadening in Fig. 5 of the main text as a computational resource in itself. Instead, the blur is better understood as a measurable dynamical consequence of neighbour-mediated spread, with excessive smoothing washing out task-relevant structure.

### Interpretation

Taken together, these ablations show that stronger diffusion-like coupling has two consistent effects: it accelerates the decay of spatial variance in the late-time field and degrades recurrent-readout accuracy. We therefore interpret the baseline reservoir as operating in a more favourable balance between spatial integration and detail preservation, whereas larger $\psi$ values move the system into an over-smoothed regime. In this sense, the useful computation of the WC–RC lies not in blur per se, but in maintaining structured spatiotemporal dynamics without erasing discriminative contrast.

## References

[1] Christensen, D. V. *et al.* 2022 roadmap on neuromorphic computing and engineering. *Neuromorphic Comput. Eng*. 1–113 (2022).

[2] Wilson, H. R. & Cowan, J. D. Excitatory and inhibitory interactions in localized populations of model neurons. *Biophys. J.* **12**, 1–24. 10.1016/S0006-3495(72)86068-5 (1972). 4332108 10.1016/S0006-3495(72)86068-5 PMC1484078

[3] Wilson, H. R. & Cowan, J. D. A mathematical theory of the functional dynamics of cortical and thalamic nervous tissue. *Kybernetik* **13**, 55–80. 10.1007/BF00288786 (1973). 4767470 10.1007/BF00288786

[4] Zúñiga-Galindo, W. A. & Zambrano-Luna, B. A. Hierarchical wilson–cowan models and connection matrices. *Entropy* 1–20 (2023). 10.3390/e25060949 PMC10297397 37372293

[5] Müller, L., Chavane, F., Reynolds, J. & Sejnowski, T. J. Cortical travelling waves: Mechanisms and computational principles. *Nat. Rev. Neurosci.*10.1038/nrn.2018.20 (2018). 29563572 10.1038/nrn.2018.20 PMC5933075

[6] Malo, J., Esteve-Taboada, J. J. & Bertalmío, M. Cortical divisive normalization from wilson-cowan neural dynamics. *J. Nonlinear Sci.*10.1007/s00332-023-10009-z (2024).

[7] dos Santos, F. P. & Verschure, P. F. M. J. Excitatory–inhibitory homeostasis and bifurcation control in the wilson–cowan model of cortical dynamics. *PLOS Comput. Biol*. 1–32 (2025). 10.1371/journal.pcbi.1012723 PMC11737862 39761317

[8] Suárez, L. E., Richards, B. A., Lajoie, G. & Mišić, B. Learning function from structure in neuromorphic networks. *Nat. Machine Intell.* **3**, 771–786. 10.1038/s42256-021-00376-1 (2021).

[9] Yamazaki, K., Vo-Ho, V.-K., Bulsara, D. & Le, N. Spiking neural networks and their applications: A review. *Brain Sci.* **12**, 863 (2022). 35884670 10.3390/brainsci12070863 PMC9313413

[10] Maass, W., Natschläger, T. & Markram, H. Real-time computing without stable states: A new framework for neural computation based on perturbations. *Neural Comput.* **14**, 2531–2560. 10.1162/089976602760407955 (2002). 12433288 10.1162/089976602760407955

[11] Jaeger, H. & Haas, H. Harnessing nonlinearity: Predicting chaotic systems and saving energy in wireless communication. *Science* **304**, 78–80. 10.1126/science.1091277 (2004). 15064413 10.1126/science.1091277

[12] Tanaka, G. *et al.* Recent advances in physical reservoir computing: A review. *Neural Netw*. 100–123 (2019). 10.1016/j.neunet.2019.03.005 30981085

[13] Kitayama, K. Guiding principle of reservoir computing based on small world network. *Sci. Rep.*10.1038/s41598-022-21235-y (2022). 36202989 10.1038/s41598-022-21235-y PMC9537422

[14] Yan, M. et al. Emerging opportunities and challenges for the future of reservoir computing. *Nat. Commun.* **15**, 1–18. 10.1038/s41467-024-45187-1 (2024). 38448438 10.1038/s41467-024-45187-1 PMC10917819

[15] Dudas, J. et al. Quantum reservoir computing implementation on coherently coupled quantum oscillators. *NPJ Quant. Inform.* **9**, 1–7 (2023).

[16] Akashi, N. et al. A coupled spintronics neuromorphic approach for high-performance reservoir computing. *Adv. Intell. Syst.* **4**, 1–13 (2022).

[17] Wanjura, C. C. & Marquardt, F. Fully non-linear neuromorphic computing with linear wave scattering. *Am. Phys. Society*. 1–18 (2024).

[18] Gabayre, S. A., Illeperuma, M., De-Silva, V. D., Shi, X. & Savel’ev, S. E. Advancements in neuromorphic computing for bio-inspired artificial vision: A review. *Neurocomputing* **653**, 131221. 10.1016/J.NEUCOM.2025.131221 (2025).

[19] Zhang, G. *et al.* Functional materials for memristor-based reservoir computing: Dynamics and applications. *Adv. Functional Mater*. 1–43 (2023).

[20] Matsukatova, A. N. et al. Combination of organic-based reservoir computing and spiking neuromorphic systems for robust and efficient pattern classification. *Adv. Intell. Syst.* **5**, 1–10 (2023).

[21] Lao, J. *et al.* Ultralow-power machine vision with self-powered sensor reservoir. *Adv. Sci*. 1–11 (2022). 10.1002/advs.202106092 PMC9130913 35285175

[22] Jaeger, H. The echo state approach to analysing and training recurrent neural networks. typeTech. Rep. numberReport 148, institutionGMD—German National Research Center for Information Technology (2001).

[23] Gepshtein, S., Pawar, A. S., Kwon, S., Savelév, S. & Albright, T. D. Spatially distributed computation in cortical circuits. *Sci. Adv*. 1–19 (2022). 10.1126/sciadv.abl5865 PMC9032974 35452288

[24] Pehle, C. *et al.* The brainscales-2 accelerated neuromorphic system with hybrid plasticity. *Front. Neurosci*. 1–21 (2022). 10.3389/fnins.2022.795876 PMC8907969 35281488

[25] Qin, H. et al. Binary neural networks: A survey. *Pattern Recognit.* **105**, 107281. 10.1016/J.PATCOG.2020.107281 (2020).

[26] Qin, H. *et al.* Bibench: benchmarking and analyzing network binarization. In *booktitleProceedings of the 40th International Conference on Machine Learning*, ICML’23 (JMLR.org, 2023).

[27] Qin, H. *et al.* Quantsr: accurate low-bit quantization for efficient image super-resolution. In *booktitleProceedings of the 37th International Conference on Neural Information Processing Systems*, NIPS ’23 (Curran Associates Inc., Red Hook, NY, USA, 2023).

[28] Qin, H. *et al.* Bipointnet: Binary neural network for point clouds. ArXiv **abs/2010.05501** (2020).

[29] Midya, R. *et al.* Artificial transneurons emulate neuronal activity in different areas of brain cortex. *Nature Communications 2025 16:1* **16**, 7289–, 10.1038/s41467-025-62151-9 (2025). 10.1038/s41467-025-62151-9 PMC12332047 40774953

[30] Deng, F., Liang, S., Qian, K., Yu, J. & Li, X. A recurrent sigma-pi-sigma neural network. *Sci. Rep.* **15**, 1–14. 10.1038/s41598-024-84299-y (2025). 39748085 10.1038/s41598-024-84299-y PMC11696508

[31] Kawai, Y., Park, J. & Asada, M. Reservoir computing using self-sustained oscillations in a locally connected neural network. *Sci. Rep*. 15532 (2023). 10.1038/s41598-023-42812-9 PMC10509144 37726352

[32] Gjorgjieva, J., Evers, J. F. & Eglen, S. J. Homeostatic activity-dependent tuning of recurrent networks for robust propagation of activity. *J. Neurosci*.3722–3734 (2016). 10.1523/JNEUROSCI.2511-15.2016 PMC4812132 27030758

[33] Guo, X., Barrett, T. D., Wang, Z. M. & Lvovsky, A. I. Backpropagation through nonlinear units for the all-optical training of neural networks. *Photon. Res*. B71–B80 (2021).

[34] LeCun, Y., Cortes, C. & Burges, C. J. MNIST handwritten digit database. *ATT Labs [Online]*. 10.1109/MSP.2012.2211477 (2010).

[35] Xiao, H., Rasul, K. & Vollgraf, R. Fashion-MNIST: A novel image dataset for benchmarking machine learning algorithms. arXiv preprint arXiv:1708.0774710.48550/arXiv.1708.07747 (2017).

